# Virtual Reality-Assisted Rehabilitation for Adolescents with Cerebral Palsy: A Systematic Review and Meta-Analysis

**DOI:** 10.3390/jcm15166248

**Published:** 2026-08-12

**Authors:** Te-Wei Chen, Yu-Ching Lin

**Affiliations:** 1 National Cheng Kung University Hospital, College of Medicine, National Cheng Kung University, Tainan 70101, Taiwan; jack89031518@gmail.com; 2 Department of Physical Medicine and Rehabilitation, National Cheng Kung University Hospital, College of Medicine, National Cheng Kung University, Tainan 70101, Taiwan; 3 Department of Physical Medicine and Rehabilitation, College of Medicine, National Cheng Kung University, Tainan 70101, Taiwan

**Keywords:** cerebral palsy, adolescent, virtual reality, rehabilitation, meta-analysis, systematic review

## Abstract

**Background/Objectives:** Cerebral palsy is a lifelong neurodevelopmental condition associated with motor impairment and activity limitation, and adolescence is a clinically important period during which functional decline or plateau may occur. Virtual reality-assisted rehabilitation may provide task-specific practice with augmented feedback and enhanced engagement. This systematic review and meta-analysis aimed to evaluate the effects of virtual reality-assisted rehabilitation on functional outcomes in adolescents aged 10 to 19 years with cerebral palsy. **Methods:** Embase, MEDLINE, and the Cochrane Central Register of Controlled Trials were searched from inception to 25 February 2026 for studies enrolling adolescents with cerebral palsy or mixed-age samples with extractable adolescent data. Two reviewers independently screened studies and extracted data. Primary domains were lower-limb/balance-related function and upper-limb function. Within-group pre–post standardised mean differences were pooled using random-effects models, and certainty was assessed using the Grading of Recommendations Assessment, Development and Evaluation approach. **Results:** Eight studies including 83 participants were eligible; seven studies including 68 participants contributed to the meta-analysis. The pooled effect was small and uncertain for lower-limb/balance-related outcomes (Hedges’ g = 0.21, 95% confidence interval −0.17 to 0.58) and moderate for upper-limb outcomes (Hedges’ g = 0.47, 95% confidence interval 0.09 to 0.84). The overall pooled estimate was positive after virtual reality-assisted rehabilitation (Hedges’ g = 0.34, 95% confidence interval 0.07 to 0.60). Certainty of evidence for both primary domains was very low. **Conclusions:** Virtual reality-assisted rehabilitation may be associated with post-intervention functional gains in adolescents with cerebral palsy, with more consistent findings for upper-limb than lower-limb/balance-related outcomes. However, the certainty of evidence was very low, and the predominantly pre–post evidence cannot establish comparative treatment efficacy. VR-assisted rehabilitation may be considered an adjunct to conventional rehabilitation to increase task-specific practice and engagement, but adolescent-focused controlled studies with harmonised outcomes and clinically meaningful follow-up are needed.

## 1. Introduction

Cerebral palsy is the most common physical disability in childhood and a leading cause of lifelong motor impairment and activity limitation [1]. It is frequently accompanied by pain and other comorbidities that compound functional burden [2]. Adolescence is a clinically important period for individuals with cerebral palsy because motor trajectories, musculoskeletal growth, participation demands, and rehabilitation priorities may change during this developmental stage. Longitudinal data suggest that gross motor function may plateau or decline during adolescence, particularly among those with greater baseline motor limitations [3,4]. Rehabilitation goals during this stage therefore extend beyond impairment to include activity, participation, and quality of life [5].

Virtual reality (VR)-assisted rehabilitation is increasingly used to deliver task-oriented practice with augmented feedback in an engaging format. Meta-analyses and overviews published in broader paediatric cerebral palsy populations have reported favourable effects of virtual reality across several motor and functional domains, but findings remain heterogeneous, and the certainty of evidence is often low [6,7,8]. However, these syntheses mainly addressed wider paediatric samples including children and adolescents together, rather than providing an adolescent-specific quantitative synthesis restricted to individuals aged 10 to 19 years. This distinction is clinically relevant because adolescents may differ from younger children in motor trajectories, secondary musculoskeletal limitations, engagement, rehabilitation goals, and participation needs. An adolescent-focused synthesis is therefore needed to clarify the currently available evidence for this age group and to inform rehabilitation planning during this developmental stage.

Accordingly, we conducted a systematic review and meta-analysis to evaluate virtual reality-assisted rehabilitation in adolescents with cerebral palsy. The primary aim was to quantify post-intervention within-group changes in lower-limb/balance-related function and upper-limb function. Secondary objectives were to synthesise other reported outcomes such as participation, health-related quality of life, pain, and motivation or adherence when available and to assess certainty of evidence using the Grading of Recommendations Assessment, Development and Evaluation (GRADE) approach.

## 2. Materials and Methods

### 2.1. Protocol and Reporting Guideline

This systematic review and meta-analysis was prospectively registered in PROSPERO, the International Prospective Register of Systematic Reviews (CRD420261326441). The review question, eligibility criteria, outcomes, and planned methods were prespecified in the registered protocol. This review was conducted and reported in accordance with the Preferred Reporting Items for Systematic Reviews and Meta-Analyses (PRISMA) 2020 statement [9]. The completed PRISMA 2020 checklist is provided in Supplementary Table S8. Ethical approval was not required because this review used data from previously published studies and did not involve new data collection from human participants, human tissue, or animals.

### 2.2. Research Question

This intervention review asked whether VR-assisted rehabilitation improves functional outcomes in adolescents aged 10 to 19 years with cerebral palsy. The primary outcome domains were lower-limb/balance-related function and upper-limb function. Secondary outcomes included overall pooled functional effects across domains, feasibility and acceptability, pain, participation, and quality of life when reported.

### 2.3. Eligibility Criteria

Eligibility criteria were prespecified using the population, intervention, comparator, outcomes, and study design framework. (1) Population: adolescents aged 10 to 19 years with cerebral palsy; mixed-age studies were eligible only if outcome data for the 10 to 19-year subgroup were separately extractable. We did not estimate adolescent-specific effects from non-extractable mixed-age samples. (2) Intervention: VR-assisted rehabilitation delivered using any platform. (3) Comparator: any comparator, including no comparator. Because the included adolescent-specific evidence was expected to be predominantly single-group pre–post evidence, the primary quantitative synthesis used intervention-arm pre–post changes to maintain a consistent effect contrast across study designs. For randomised or non-randomised controlled studies, controlled-arm findings were examined separately to assess the feasibility of between-group sensitivity analysis but were not pooled together with within-group pre–post effects. (4) Outcomes: functional outcomes mapped to prespecified domains (lower-limb/balance-related function; upper-limb function). Other outcomes, such as participation, health-related quality of life, pain, motivation, adherence, feasibility, and acceptability, were summarised narratively when reported. (5) Study design: randomised or non-randomised controlled studies, single-group pre–post studies, and single-case experimental designs.

### 2.4. Information Sources and Search Strategy

We searched Embase (Embase.com, Elsevier, Amsterdam, The Netherlands), MEDLINE (Ovid), and the Cochrane Central Register of Controlled Trials (CENTRAL) from inception to 25 February 2026 (date last searched), without language or publication-status restrictions in the electronic searches; age eligibility criteria were applied during screening and data extraction. Search terms combined controlled vocabulary and free-text terms for cerebral palsy, adolescence, VR, exergaming, motor rehabilitation, and related constructs. The full search strategies for all databases are provided in Supplementary Table S7. Records indexed with non-English titles or abstracts were not excluded a priori. Where bibliographic databases provided English titles or abstracts, these were used for initial screening; if full-text assessment of a potentially eligible non-English report had been required, machine-assisted translation would have been used.

### 2.5. Study Selection

Records were imported into EndNote 2025 (Clarivate) for deduplication. Duplicates were identified using automated cross-checking of fields including title, author, year, and Digital Object Identifier (DOI), followed by manual review to ensure accuracy. Two reviewers independently screened titles/abstracts and then assessed full texts against prespecified criteria. Disagreements were resolved by discussion and consensus, with adjudication by the senior author when needed. Reasons for full-text exclusion were recorded. Studies were eligible for quantitative synthesis when outcome data mapped to the prespecified functional domains and were reported with sufficient information for effect size estimation; otherwise, results were synthesised narratively.

### 2.6. Data Extraction

Two reviewers independently extracted study characteristics, participant information, intervention details, comparator characteristics when applicable, and outcome data using a standardised, piloted extraction form. Intervention details included VR modality/platform, device type, setting, supervision, session duration, frequency, total intervention duration, and co-interventions. For meta-analysis, we extracted sample sizes and pre- and post-intervention means and standard deviations (SDs) at the prespecified post-intervention time point. When more than one potentially relevant outcome was reported within a functional domain, one prespecified pooled outcome was selected per study according to domain relevance, clinical interpretability, and availability of extractable pre–post data; the rationale for outcome selection and directionality is documented in Supplementary Table S5. When change-score SDs were required, we used the within-participant pre–post correlation coefficient (r) when it could be derived from adolescent participant-level or matched-pair data. When r could not be derived from the available data, we used r = 0.50 in the primary analysis and documented the assumption in the extraction and derivation logs. Discrepancies were resolved by discussion and consensus, with adjudication by the senior author when needed.

### 2.7. Risk of Bias Assessment

Risk of bias was assessed independently by two reviewers using design-appropriate tools aligned with the extracted data. Single-group pre–post studies were appraised using the National Institutes of Health (NIH) Quality Assessment Tool for Before–After (Pre–Post) Studies with No Control Group [10]. Single-case experimental designs were appraised using the Risk of Bias in N-of-1 Trials (RoBiNT) tool [11]. Non-randomised controlled studies were appraised using Risk Of Bias In Non-randomised Studies—of Interventions (ROBINS-I) [12]. Discrepancies were resolved by consensus, with adjudication by the senior author when needed (Supplementary Table S1).

### 2.8. Outcomes

Outcomes were grouped a priori by clinical and functional domain. The primary domains were (i) lower-limb/balance-related function and (ii) upper-limb function. These domains were selected because they represented the functional endpoints most consistently reported across adolescent-specific VR rehabilitation studies and were clinically relevant to rehabilitation planning in cerebral palsy. Outcome measures were mapped to domains and harmonised so that higher values indicated better function; where instruments were scored in the opposite direction, effect directions were reversed. Because different instruments were used to represent related but non-identical constructs within each domain, pooled effects were interpreted as standardised domain-level functional change rather than instrument-specific treatment effects. The primary time point was the end of the intervention period. Domain mapping, selected pooled outcomes, and directionality are documented in Supplementary Table S5. Secondary outcomes included feasibility, acceptability, adherence, motivation, participation, health-related quality of life, pain, and adverse events when reported. These outcomes were summarised narratively because they were inconsistently reported and were not sufficiently comparable for quantitative synthesis.

### 2.9. Data Synthesis and Statistical Analysis

We synthesised continuous outcomes using standardised mean differences (Hedges’ g) [13]. For each study, intervention-arm within-group pre–post change scores were calculated and pooled using a random-effects model [14], with two-sided *p* < 0.05 considered statistically significant. This approach was selected to maintain a consistent effect contrast across the predominantly uncontrolled adolescent-specific evidence base. Therefore, the pooled estimates should be interpreted as pre–post associations after VR-assisted rehabilitation rather than definitive between-group treatment effects. Effect directions were harmonised so that higher values indicated better function. Heterogeneity was quantified using I^2^ [15]. Prespecified subgroup analyses were conducted by functional domain, intervention duration, and device type. Pooled effects were reported using random-effects estimates. For functional-domain subgroup comparisons, the fixed-effect Q_between test from the CMA subgroup output was used to evaluate subgroup differences, and the findings were interpreted cautiously given the small number of studies and heterogeneity. Sensitivity analyses included leave-one-out analyses and an inverse variance heterogeneity (IVhet) model using MetaXL v5.3. In response to uncertainty in imputed change-score SDs, post hoc sensitivity analyses were performed by varying the assumed within-participant pre–post correlation coefficient for studies requiring imputed r values from r = 0.50 to r = 0.25 and r = 0.75 while retaining empirically derived correlations for studies with adolescent participant-level or matched-pair data (Supplementary Table S9). The feasibility of pooled between-group sensitivity analysis among controlled studies was assessed separately and is reported in Supplementary Table S10. Additional potential sources of heterogeneity, including CP subtype, GMFCS/MACS level, VR platform/interface, intervention dose, setting, and outcome-measure responsiveness, were examined descriptively to determine whether further formal subgroup analyses were feasible (Supplementary Table S11). Small-study effects and publication bias were examined using funnel plots, Egger’s test [16], trim-and-fill [17], and Doi plots with the Luis Furuya-Kanamori (LFK) index [18]. Analyses were primarily conducted in Comprehensive Meta-Analysis (CMA) v3.3.070 (Windows GUI), with supplementary analyses performed in MetaXL v5.3 (Excel add-in). No novel computer code was developed.

### 2.10. Certainty of Evidence

We rated certainty of evidence for the primary outcome domains using the Grading of Recommendations Assessment, Development and Evaluation (GRADE) approach to rate certainty as high, moderate, low, or very low [19,20]. Certainty was assessed across risk of bias, inconsistency, indirectness, imprecision, and publication bias. Because pooled estimates primarily reflected within-group pre–post change, we prespecified very serious concerns for residual confounding when downgrading for risk of bias.

### 2.11. Lived Experience Involvement

People with cerebral palsy and caregivers were not directly involved in formulating the review question, selecting outcomes, interpreting findings, or writing this manuscript. The review was designed and interpreted by the author team, including a rehabilitation physician with clinical expertise in cerebral palsy.

## 3. Results

### 3.1. Study Selection

**Figure 1:**
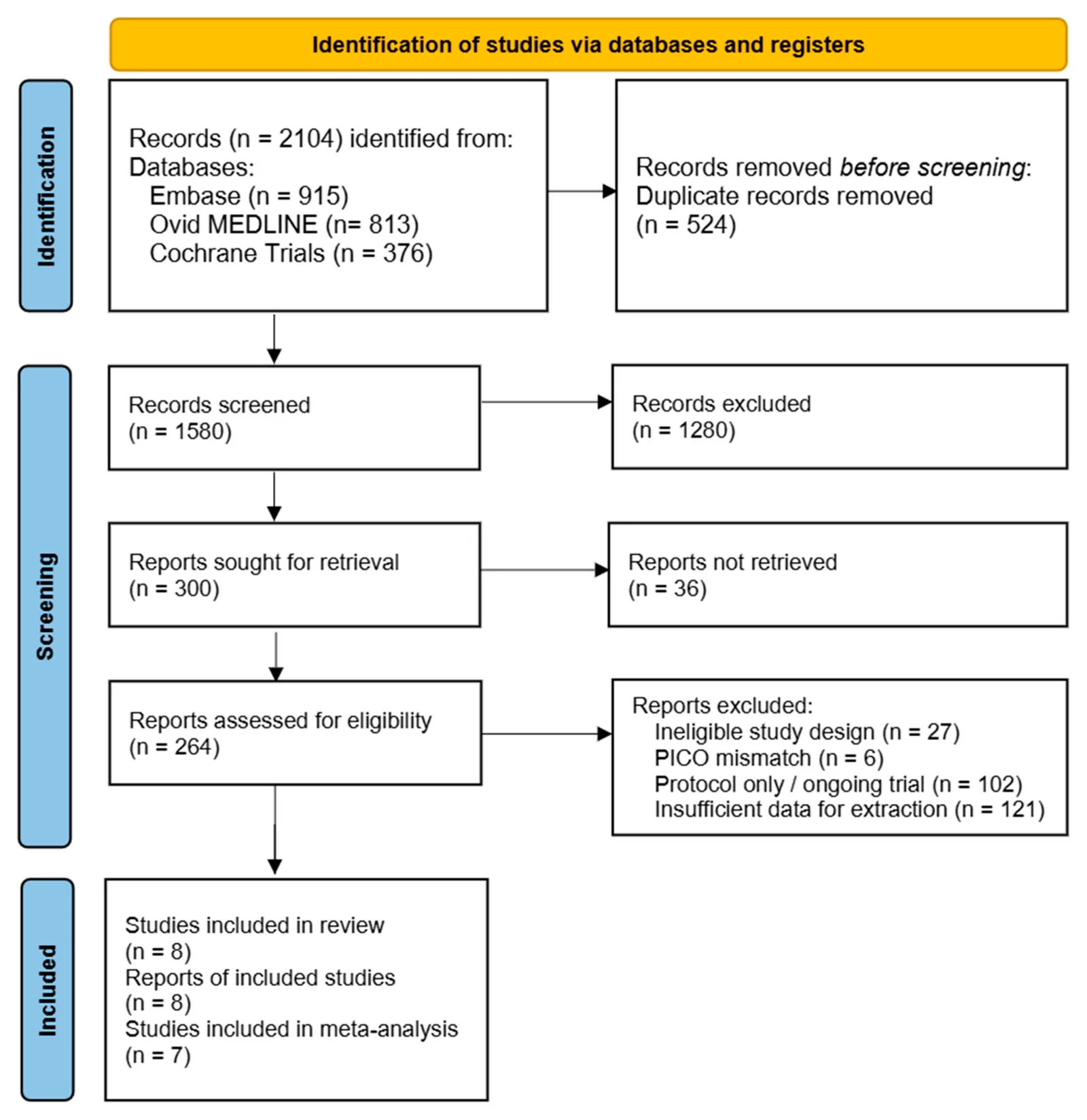
Preferred Reporting Items for Systematic Reviews and Meta-Analyses (PRISMA) 2020 flow diagram of study selection. The diagram summarises record identification from Embase, Ovid MEDLINE, and Cochrane Trials; removal of duplicates; title/abstract screening; full-text assessment; reasons for exclusion; and final inclusion in the systematic review and meta-analysis.

Figure 1 summarises the study selection process. The database search identified 2104 records (Embase, *n* = 915; MEDLINE, *n* = 813; CENTRAL, *n* = 376). After removal of 524 duplicates, 1580 records were screened by title and abstract, and 300 reports were sought for full-text assessment; 36 reports could not be retrieved. Of the 264 full-text reports assessed, 256 were excluded, most commonly due to insufficient data for extraction (*n* = 121), protocol-only or ongoing trial status (*n* = 102), or ineligible study design (*n* = 27); other exclusions were due to ineligible population (*n* = 3), intervention (*n* = 2), or outcomes (*n* = 1) (Supplementary Table S6). Eight studies met the inclusion criteria for qualitative synthesis, and seven were included in the meta-analysis. No additional eligible studies were identified through reference list screening.

### 3.2. Study Characteristics

Table 1 summarises the eight included studies (total *n* = 83; sample sizes 5–28) [21,22,23,24,25,26,27,28]. Mean participant age was in early to mid-adolescence, approximately 11–15 years. Among the eight studies, there were five single-group pre–post studies, one randomised controlled trial, one non-randomised controlled study, and one single-case experimental design. Participants predominantly had spastic cerebral palsy, most commonly bilateral involvement; functional classification was generally in the mild-to-moderate range when reported, including Gross Motor Function Classification System (GMFCS) levels I–III and Manual Ability Classification System (MACS) levels I–III. All interventions used non-immersive VR-assisted platforms, including commercially available exergaming systems and rehabilitation-oriented platforms, such as sensor/biofeedback or haptic interfaces and treadmill/robotic gait systems, typically delivered multiple times per week over several weeks. For each study contributing to the meta-analysis, the prespecified pooled outcome is indicated by a dagger (†) in Table 1. Adverse events were infrequently reported across studies, and no serious adverse events were described.

### 3.3. Effects of Interventions

**Figure 2:**
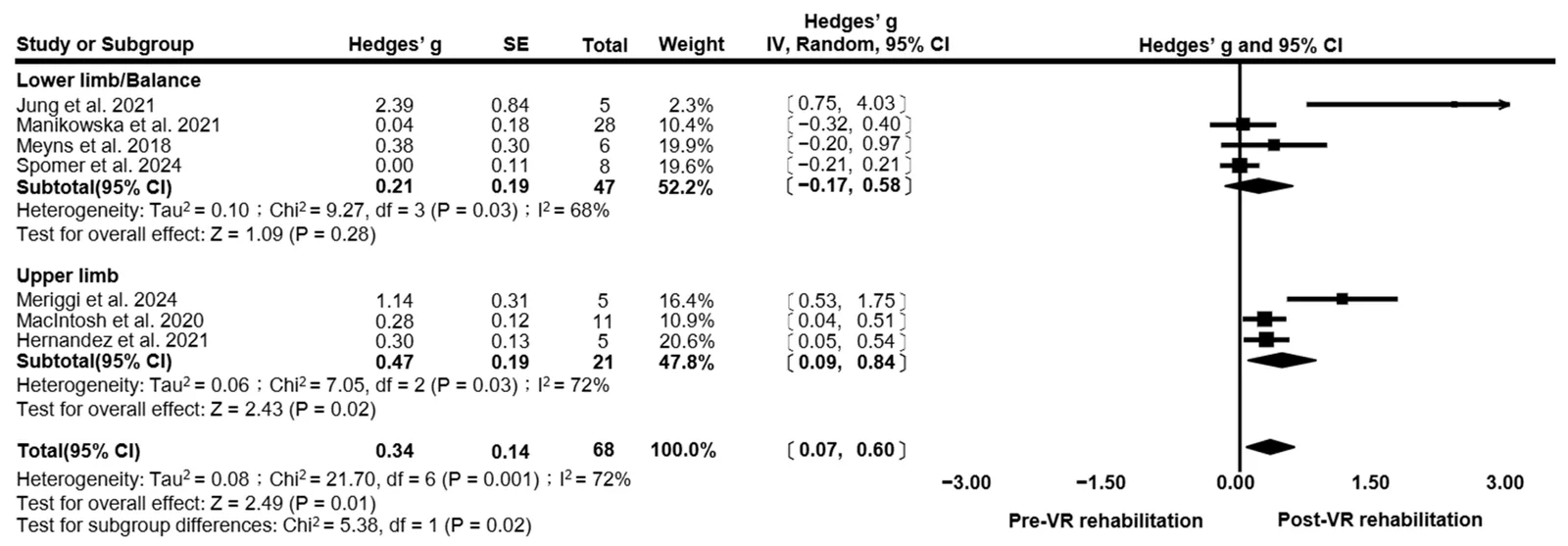
Forest plot of within-group pre–post functional change after virtual reality-assisted rehabilitation, stratified by lower-limb/balance-related and upper-limb domains [21,22,23,25,26,27,28]. Effect sizes are presented as Hedges’ g with 95% confidence intervals using random-effects models. Positive values indicate greater post-intervention improvement. Subgroup pooled estimates and the overall pooled estimate are shown. The fixed-effect Q_between test for subgroup differences compares pooled effects between lower-limb/balance-related and upper-limb domains. **Abbreviation:** VR, virtual reality.

Figure 2 shows pooled within-group effects at post-intervention. For lower-limb/balance-related outcomes (four studies; *n* = 47), the pooled effect was small and statistically uncertain (Hedges’ g = 0.21, 95% confidence interval −0.17 to 0.58; I^2^ = 68%). For upper-limb outcomes (three studies; *n* = 21), the pooled estimate was moderate and positive (Hedges’ g = 0.47, 95% confidence interval 0.09 to 0.84; I^2^ = 72%). Across all pooled outcomes (seven studies; *n* = 68), the overall pooled estimate was positive after VR-assisted rehabilitation (Hedges’ g = 0.34, 95% confidence interval 0.07 to 0.60; I^2^ = 72%). The fixed-effect Q_between test suggested a difference between the prespecified functional domains (χ^2^ = 5.38, df = 1; *p* = 0.02), although this comparison should be interpreted cautiously given the small number of studies and substantial within-domain heterogeneity. Secondary outcomes were reported inconsistently across studies and were therefore not quantitatively pooled. These included patient-reported performance or activity measures, participation or activity-setting experiences, hand-use questionnaires, pain, health-related quality of life, feasibility, adherence, and adverse events, depending on the study. One included study was not pooled because its outcomes, including feasibility, pain, and health-related quality of life, were not sufficiently comparable with the prespecified functional domains and were therefore summarised narratively. In the feasibility study by Cardenas et al. [24], inpatient cycling-based exergames following lower-extremity orthopaedic surgery were feasible and enjoyable, with all feasibility indicators met. Patient-reported outcomes included the Faces Pain Scale–Revised (FPS-R), Patient-Reported Outcomes Measurement Information System Pain Interference (PROMIS-PI), and KIDSCREEN-27; between-group analyses favoured the exergame case group for KIDSCREEN-27 total and psychological well-being scores, whereas pain outcomes were exploratory and variable. No serious adverse events were reported.

### 3.4. Risk of Bias Within Studies

**Figure 3:**
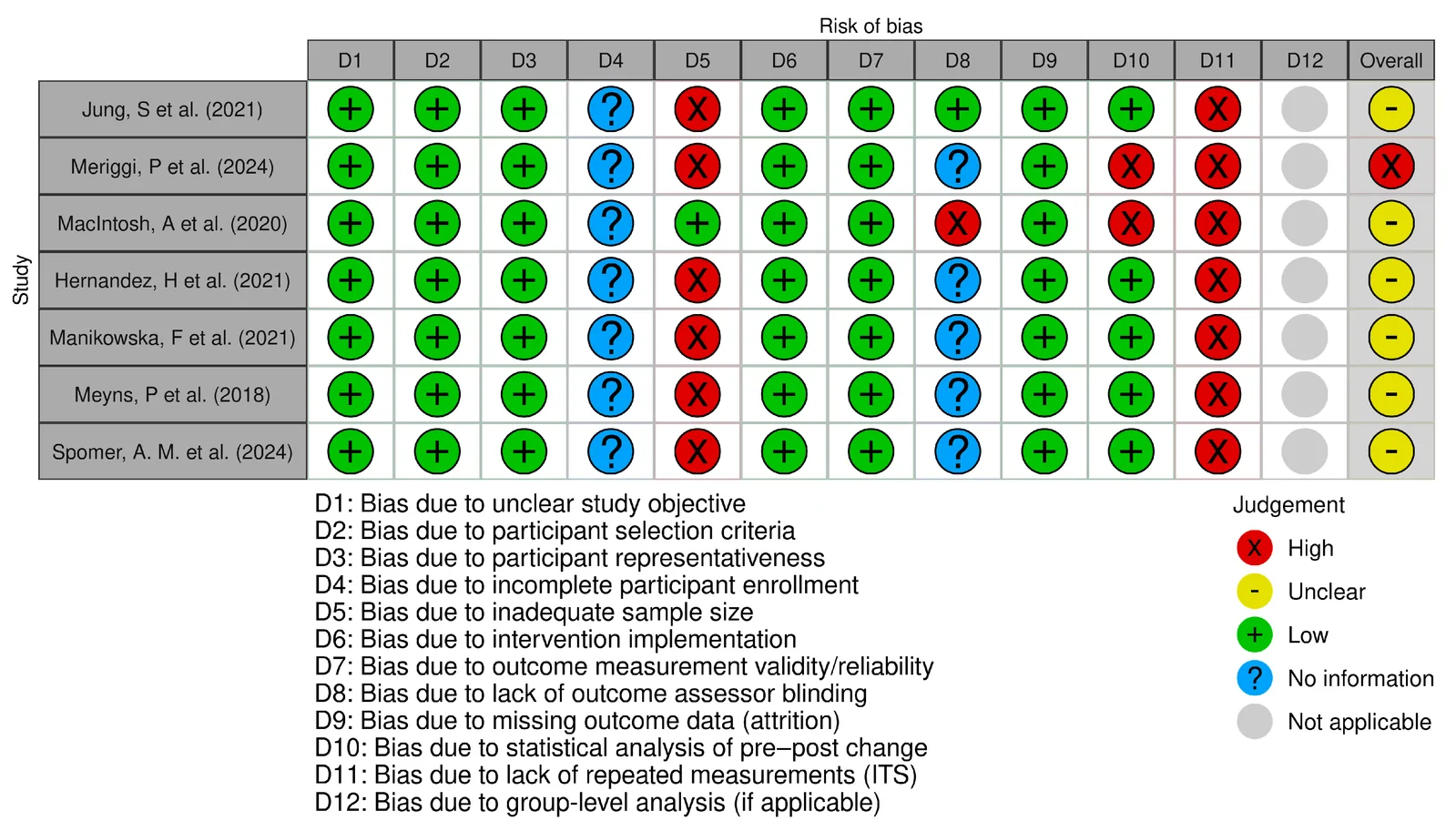
Summary visualisation of risk-of-bias assessments for studies included in the meta-analysis [21,22,23,25,26,27,28]. Traffic-light judgements are shown for each pooled study across prespecified domains (D1–D12) and for the overall study-level judgement, using a harmonised NIH-based domain framework for visualisation. Symbols indicate low risk, high risk, unclear risk, no information, or not applicable, as defined in the figure. Domain definitions are provided within the figure. Tool-specific risk-of-bias assessments for all included studies are reported in Supplementary Table S1.

Figure 3 summarises risk-of-bias assessments for studies included in the meta-analysis; tool-specific judgements for all included studies are provided in Supplementary Table S1. Across the included studies, the overall risk of bias was commonly influenced by incomplete reporting for several domains and by limitations inherent to uncontrolled pre–post designs. Domains related to outcome measurement were generally judged at lower risk when validated instruments were used.

### 3.5. Additional Analyses

In duration subgroup analyses, interventions lasting ≥6 weeks showed a pooled effect of Hedges’ g = 0.39 (95% confidence interval 0.04 to 0.73; I^2^ = 77%; six studies; *n* = 47), whereas the short-duration subgroup (<6 weeks) showed no clear effect (Hedges’ g = 0.28, 95% confidence interval −0.43 to 0.99; one study; *n* = 11); the subgroup difference was not statistically significant (χ^2^ = 0.36, df = 1; *p* = 0.55) (Supplementary Figure S1). In device subgroup analyses, commercial platforms showed a larger pooled effect (Hedges’ g = 0.73, 95% confidence interval 0.01 to 1.45; I^2^ = 80%; two studies; *n* = 11) than rehabilitation-specific devices (Hedges’ g = 0.27, 95% confidence interval −0.01 to 0.55; I^2^ = 72%; five studies; *n* = 57), without evidence of a subgroup difference (χ^2^ = 2.06, df = 1; *p* = 0.15) (Supplementary Figure S2). Leave-one-out analyses suggested that the overall pooled estimate was not driven by any single study (Hedges’ g range 0.23–0.43) (Supplementary Figure S3). Sensitivity analyses varying the imputed pre–post correlation coefficient for Jung et al. [23] and Manikowska et al. [26] from r = 0.50 to r = 0.25 and r = 0.75 did not materially change the interpretation. Across these assumptions, lower-limb/balance-related estimates remained statistically uncertain, upper-limb estimates remained positive, and overall pooled estimates remained positive; the fixed-effect Q_between test for functional-domain subgroup differences was also stable (*p* = 0.020–0.021) (Supplementary Table S9). A formal pooled between-group sensitivity meta-analysis was not feasible. Among controlled studies, Jung et al. [23] was the only randomised controlled trial with a compatible primary functional outcome, whereas Cardenas et al. [24] was a non-randomised feasibility study reporting feasibility, pain, and health-related quality-of-life outcomes rather than the prespecified functional domains (Supplementary Table S10). Additional descriptive exploration of potential heterogeneity sources suggested that further formal subgroup meta-analyses by CP subtype, GMFCS/MACS level, VR platform/interface, dose, setting, or outcome-measure responsiveness were not appropriate because of the small number of studies, sparse data within categories, overlapping classifications, and heterogeneous outcome instruments (Supplementary Table S11). Across all pooled studies (k = 7), small-study effects and publication-bias diagnostics suggested asymmetry (Egger’s test *p* = 0.044; LFK index = 3.59), whereas trim-and-fill did not impute additional studies (0 imputed) (Supplementary Figure S4). These diagnostics should be interpreted cautiously given the small number of studies and their application across mixed outcome domains. Because trial registries and grey-literature sources were not searched, unpublished or ongoing studies may have been missed, and the observed asymmetry may reflect publication bias, selective reporting, small-study effects, or heterogeneity rather than a single mechanism. In an IVhet model sensitivity analysis, the overall pooled estimate was Hedges’ g = 0.215 (95% confidence interval −0.072 to 0.502).

### 3.6. Certainty of Evidence

Certainty of evidence for both primary outcome domains was rated as very low (Table 2). Certainty was downgraded primarily for very serious risk of bias related to residual confounding in within-group pre–post evidence and further downgraded for inconsistency and imprecision. For lower-limb/balance-related function, certainty was additionally downgraded for indirectness because different instruments were used to represent the domain. Possible small-study effects were also noted in overall publication-bias diagnostics, although these findings were interpreted cautiously given the small number of studies and mixed outcome domains.

## 4. Discussion

### 4.1. Principal Findings

To our knowledge, this is the first quantitative synthesis focused specifically on adolescents with cerebral palsy. Our meta-analysis suggests that VR-assisted rehabilitation may be associated with small-to-moderate pre–post functional changes in this age group, but these findings should be considered suggestive rather than conclusive because the certainty of evidence is very low. At post-intervention, pooled estimates varied by prespecified domain: upper-limb outcomes showed a moderate positive estimate (Hedges’ g = 0.47), whereas lower-limb/balance-related outcomes showed a small and statistically uncertain estimate (Hedges’ g = 0.21), with substantial heterogeneity in both domains. The overall pooled estimate across studies was small (Hedges’ g = 0.34). The fixed-effect Q_between test suggested a difference between functional domains, but this comparison should be interpreted cautiously because it was based on a small number of heterogeneous studies. Because the pooled estimates were based on intervention-arm pre–post change scores, they should not be interpreted as definitive evidence of comparative treatment efficacy.

The direction of the overall estimate was robust to leave-one-out analysis and to alternative IVhet modelling, and the r-sensitivity analysis did not materially change the interpretation. Most included interventions used non-immersive, game-based platforms, consistent with current clinical practice. Adverse events were rarely reported, and no serious adverse events were described. One controlled feasibility study reporting primarily feasibility, pain, and health-related quality-of-life outcomes was not pooled and was synthesised narratively, highlighting the limited adolescent-specific evidence beyond functional motor domains. Engagement may be an important mediator during adolescence, but evidence linking engagement to functional gains in adolescent cerebral palsy remains indirect.

### 4.2. Interpretation and Comparison with Prior Literature

Several factors may explain the modest and heterogeneous effects observed. First, intervention doses varied substantially; in paediatric neurorehabilitation, motor gains are dose-dependent, and higher treatment intensity is associated with better outcomes [29]. In VR-focused syntheses, dose–response patterns may be non-linear, with suggested optimal benefits around 30 to 40 h of exposure [8]. Second, adolescents with cerebral palsy may have more established compensatory movement patterns and secondary musculoskeletal limitations than younger children, potentially narrowing the scope for neuroplasticity-driven gains [1,4]. Third, outcome measurement heterogeneity, particularly for gait and balance, may have contributed to indirectness, as instruments vary and responsiveness evidence is limited [30,31]. Additional clinical and methodological sources of heterogeneity included CP subtype, GMFCS/MACS level, intervention setting, supervision, platform/interface characteristics, treatment dose, and whether the VR task primarily targeted upper-limb practice, standing balance, gait, or cycling-based exercise.

These factors may partly explain why estimates appeared more consistent for upper-limb outcomes. Upper-limb VR tasks often permit high-repetition, visually guided practice in relatively stable seated or supported postures, allowing feedback to be directed toward task-specific hand or arm performance. In contrast, lower-limb and balance interventions require concurrent postural control, weight shifting, anticipatory and reactive balance strategies, and gait-related coordination, making the observed response more dependent on baseline motor severity, environmental constraints, supervision, and platform characteristics. Prior syntheses similarly report less consistent and more heterogeneous effects for gait and postural control, generally with low certainty [6,32]. Measurement responsiveness may also differ across domains. Upper-limb measures such as dexterity or task-performance tests may capture short-term practice-related changes more readily, whereas gait speed, balance scales, and gross motor measures can be influenced by ceiling effects, baseline ambulatory status, musculoskeletal constraints, and differences in the construct measured by each instrument [30,31]. Intervention design may further contribute to domain-specific uncertainty. Commercial exergames can enhance motivation and practice intensity, but they may not provide the same task-specific progression, dynamic postural support, therapist-adjusted feedback, or impairment-specific targeting as rehabilitation-designed systems. This may be particularly relevant for lower-limb and balance training, where safety, postural alignment, and progressive challenge are central to treatment delivery.

Three reviews in the literature are particularly relevant when interpreting the present findings. Li et al. synthesised 41 randomised controlled trials of VR interventions in children and adolescents with cerebral palsy and reported beneficial effects across gait, balance, gross motor function, activities of daily living, and hand function; however, their review addressed a broad paediatric population rather than adolescents and pooled between-group comparisons [6]. Fang et al. conducted an overview of 16 systematic reviews/meta-analyses, identified substantial overlap among the primary studies, and found that the methodological quality of the literature was predominantly low or very low, highlighting uncertainty in the broader evidence base [7]. AlSoqih et al. evaluated children with cerebral palsy aged 4 to 18 years, included 16 studies in qualitative synthesis, and reported a moderate overall effect in a randomised controlled trial-based meta-analysis; however, their review used a wide paediatric framework and a different analytic contrast from the present synthesis [8]. In contrast, our review was designed specifically to address adolescents aged 10 to 19 years, allowing inclusion of mixed-age studies only when adolescent data were separately extractable, and it characterised adolescent-specific outcome patterns across prespecified functional domains. In this narrower clinical context, upper-limb outcomes appeared more consistent than lower-limb/balance-related outcomes, whereas certainty of evidence remained very low. These findings therefore complement the literature by clarifying what the currently available evidence supports regarding VR-assisted rehabilitation for adolescents with cerebral palsy.

### 4.3. Clinical Implications

Given the very low certainty of evidence, these findings should be interpreted cautiously. The present findings suggest that VR-assisted rehabilitation may be considered an adjunct to conventional therapy for adolescents with cerebral palsy, particularly when the aim is to increase task-specific practice and engagement. A recent synthesis reported a favourable safety profile, with low rates of adverse events and no serious side effects [8]. In practice, selection of the platform and tasks should align with the targeted functional domain, such as upper-limb versus gait/balance function, and with the individual’s baseline abilities, with attention to fatigue, supervision, and accessibility. Because dose and adherence may influence outcomes, structured programmes with sufficient cumulative exposure and progression should be prioritised [33]. For lower-limb and balance training, combining VR with standing balance or gait training may help manage task complexity and reduce variability across settings [34,35]. Adolescent-reported measures of participation and quality of life should also be included alongside functional endpoints [36,37].

### 4.4. Strengths and Limitations

Strengths of this systematic review and meta-analysis include prospective registration, PRISMA 2020 reporting, adolescent-specific eligibility criteria, and prespecified functional domains with harmonised directionality. We used design-appropriate risk-of-bias tools aligned with a within-group pre–post synthesis approach and documented all data derivations and imputations transparently. Sensitivity analyses, including leave-one-out analysis, IVhet modelling, and r-sensitivity analyses, supported the stability of the direction of the overall pooled estimate, while feasibility assessments clarified why additional between-group and subgroup meta-analyses were not performed.

Several limitations should be considered. Most included studies were uncontrolled or contributed intervention-arm pre–post data, and many risk-of-bias domains were rated as unclear or high risk; therefore, causal attribution is limited. Observed changes may reflect VR-assisted rehabilitation, but they may also be influenced by natural maturation, adolescent growth-related changes, regression to the mean, Hawthorne effects, repeated-testing or practice effects, co-interventions, and differences in usual care. Regression to the mean is particularly relevant in pre–post designs because part of the observed change may reflect statistical tendency rather than treatment response [38,39]. Long-term follow-up data were limited or absent across the included studies; therefore, the sustainability of post-intervention changes and their transfer to everyday activity, participation, and quality of life remain uncertain. This is particularly important in adolescents with cerebral palsy because rehabilitation benefits need to be maintained during ongoing growth, changing activity demands, and transition toward adult roles. Substantial heterogeneity and small sample sizes reduced precision and constrained subgroup and publication-bias assessments. Although we descriptively examined potential sources of heterogeneity, including CP subtype, GMFCS/MACS level, VR platform/interface, intervention dose, setting, and outcome-measure responsiveness, further formal subgroup meta-analyses were not appropriate because data were sparse within categories and classifications overlapped across studies (Supplementary Table S11). For lower-limb/balance-related function, indirectness was a concern because different instruments were used to represent the same domain. Publication-bias diagnostics suggested possible small-study effects (Egger’s test *p* = 0.044; LFK index = 3.59), but interpretation is limited by the small number of studies and mixed outcome domains. Because we did not search trial registries, conference proceedings, or other grey-literature sources, unpublished, ongoing, or incompletely reported studies may have been missed. Therefore, the observed funnel and Doi plot asymmetry may reflect publication bias, selective reporting, small-study effects, or heterogeneity rather than a single mechanism, and the pooled estimates may overestimate the true effect. Prior reviews similarly reported low certainty and substantial heterogeneity [6,7]. Safety reporting is inconsistent in the VR rehabilitation literature, and adverse events may be under-reported [40]. Accordingly, current findings should be interpreted as suggestive pre–post associations rather than conclusive evidence of comparative efficacy, and adequately powered controlled studies using harmonised core outcomes, standardised intervention reporting, longer-term follow-up, and standardised harms and adverse-event reporting are needed to confirm the magnitude, sustainability, and clinical relevance of effects.

### 4.5. Future Research

Future research should prioritise adequately powered, adolescent-focused controlled trials with prospectively defined comparator conditions. Intervention dose, progression rules, co-interventions, therapist involvement, adherence, and fidelity should be reported using structured rehabilitation intervention reporting frameworks, such as the TIDieR-Rehab checklist [41]. Trials should prespecify clinically meaningful post-intervention and longer-term follow-up time points to assess maintenance of gains, transfer to everyday activity and participation, and durability across adolescent growth and transition. Outcome selection should be standardised through harmonised, International Classification of Functioning, Disability and Health-informed core outcome domains for children and youth with cerebral palsy [36], with routine inclusion of adolescent-reported participation and quality-of-life outcomes, including age-appropriate cerebral palsy-specific patient-reported measures [42], alongside clinician-measured functional endpoints.

Future work should compare technology categories and delivery modes, including commercial exergames, rehabilitation-designed systems, home-based platforms, and supervised clinical programmes, and should test whether combining VR with conventional gait and balance training improves the consistency and transferability of lower-limb outcomes [8,43]. To strengthen causal inference, trials should incorporate appropriate control conditions guided by structured approaches to control selection [44], including attention-matched or dose-matched comparators when feasible, so that VR-specific effects can be distinguished from additional therapy time, therapist attention, usual care, and expectancy effects. Analytic strategies should be prespecified to minimise bias from maturation, regression to the mean, attrition, co-interventions, and repeated-testing effects [45]. Harms and tolerability should be reported using standardised definitions and reporting frameworks [46,47], including adverse events, falls, fatigue, pain exacerbation, cybersickness, discomfort, adherence barriers, and reasons for withdrawal.

## 5. Conclusions

In adolescents with cerebral palsy, VR-assisted rehabilitation may be associated with small-to-moderate post-intervention functional changes, with more consistent findings for upper-limb outcomes than for lower-limb/balance-related outcomes. However, the certainty of evidence was very low, mainly because the available evidence was limited by uncontrolled or intervention-arm pre–post designs, small sample sizes, heterogeneity, and imprecision. These findings should therefore be interpreted as suggestive pre–post associations rather than conclusive evidence of comparative efficacy. VR-assisted rehabilitation may be considered an adjunct to conventional rehabilitation to increase task-specific practice and engagement, but definitive recommendations require high-quality adolescent-focused controlled studies with harmonised outcome measures, standardised intervention and harms reporting, and clinically meaningful follow-up.

## Figures and Tables

**Table 1:** Characteristics of included studies and prespecified pooled outcomes.

Author	Study Design	Sample Size	Age Mean (SD)	Sex (M/F)	Intervention	Platform	CP Subtype	GMFCS/MACS	Frequency and Duration	Outcomes
Jung et al. (2021) [23]	RCT	IG: 5 CG: 5	IG: 12.80 (1.60) CG: 12.00 (2.53)	IG: 3/2 CG: 2/3	IG: VR-based balance training (Kinect Sports) + Conventional PT CG: Conventional PT	Xbox 360 Kinect	Spastic bilateral (10)	IG GMFCS: level I (2), level II (3); MACS: level I (2), level II (3) CG GMFCS: level I (3), level II (2); MACS: level I (3), level II (2)	IG: 40 min (VR) + 40 min (PT)/session, 3 sessions/week for 6 weeks CG: 40 min (PT)/session, 3 sessions/week for 6 weeks	PBS † SCALE GAITRite
Meriggi et al. (2024) [27]	Single-group Pre–post Design	Total: 5 (10–19 y subset)	11.40 (2.07)	5/0	VR-based upper limb training (VITAMIN games)	PC + Kinect v2	Spastic unilateral (5)	GMFCS: level I (5) MACS: level II (2), level III (3)	45 min (VR)/session, 1 session/week for 10 weeks	MA2 † MAS PROM/AROM ABILHAND-Kids
MacIntosh et al. (2020) [22]	SCED	Total: 11 (10–19 y subset)	12.92 (2.41)	4/7	Home-based biofeedback upper-limb training (Dashy Square)	Laptop + Myo Armband (EMG/IMU)	Spastic unilateral (7); Mixed (4)	GMFCS: N/A MACS: level I (6), level II (5)	17 min (VR)/session, 4 sessions/week for 4 weeks	BBT † Active wrist extension Grip strength AHA COPM SEAS
Cardenas et al. (2021) [24]	Non-randomised Controlled Trial (NRCT)	IG: 5 CG: 5	IG: 14.00 (2.80) CG: 13.80 (2.90)	IG: 3/2 CG: 3/2	IG: Inpatient cycling exergaming (Liberi) + Conventional PT CG: Conventional PT	Stationary Bike + PC	Spastic bilateral (9); Spastic unilateral (1)	IG GMFCS: level II (4), level III (1); MACS: N/A CG GMFCS: level II (2), level III (3); MACS: N/A	IG: 30 min (VR)/session, 5 sessions/week for 3 weeks CG: 5–10 min (Cycling)/session, 1–5 sessions/week for 3 weeks	Feasibility FPS-R PROMIS-PI KIDSCREEN-27 Heart rate
Hernandez et al. (2021) [25]	Single-group Pre–post Design	Total: 5 (10–19 y subset)	11.93 (2.09)	2/3	Haptic-feedback upper-limb gaming (Custom games)	PC + Novint Falcon (Haptic device)	Spastic unilateral (5)	GMFCS: N/A MACS: level I (2), level II (1), N/A (2)	60 min (VR)/session, 1 session/week for 12 weeks	QUEST † COPM Grip strength CHEQ
Manikowska et al. (2021) [26]	Single-group Pre–post Design	Total: 28	15.20 (2.00)	18/10	Robot-assisted gait training with VR feedback	Robotic Gait Trainer/Treadmill + VR System	Spastic bilateral (28)	GMFCS: level I (11), level II (8), level III (5), level IV (4) MACS: N/A	30 min (VR Treadmill)/session, 5 sessions/week for 6 weeks	GMFM-88 † 6MWT 10MWT
Meyns et al. (2018) [21]	Single-group Pre–post Design	Total: 6 (10–19 y subset)	12.80 (2.17)	5/1	Home-based balance training (Kinect Sports)	Xbox One + Kinect	Spastic bilateral (6)	GMFCS: level II (6) MACS: N/A	30 min (VR)/session, 5 sessions/week for 6 weeks	PBS † sdMoS
Spomer et al. (2024) [28]	Single-group Pre–post Design	Total: 8	14.25 (2.12)	7/1	Treadmill walking with biofeedback	Treadmill + Biofeedback System	Not specified (8)	GMFCS: level I (2), level II (4), level III (2) MACS: N/A	20 min (VR/Biofeedback)/session, 4 sessions total	Gait speed † GDI MEAN_SOL_STANCE Step Length/Stance Time

**Footnotes:** † Prespecified primary outcome used for meta-analysis (one per study). Cardenas et al. was not meta-analysed because its outcomes did not match the predefined functional domains. For randomised controlled trials, only intervention-group pre–post data were pooled to harmonise effect size estimation across predominantly single-group pre–post studies; control-group findings are reported descriptively. **Abbreviations:** 10MWT, 10-Meter Walk Test; 6MWT, 6-Minute Walk Test; ABILHAND-Kids, ABILHAND-Kids questionnaire; AHA, Assisting Hand Assessment; AROM, active range of motion; BBT, Box and Block Test; CG, control group; CHEQ, Children’s Hand-use Experience Questionnaire; COPM, Canadian Occupational Performance Measure; CP, cerebral palsy; EMG, electromyography; FPS-R, Faces Pain Scale–Revised; GAITRite, GAITRite^®^ walkway system for spatiotemporal gait analysis; GDI, Gait Deviation Index; GMFCS, Gross Motor Function Classification System; GMFM-88, Gross Motor Function Measure-88; IG, intervention group; IMU, inertial measurement unit; MACS, Manual Ability Classification System; MAS, Modified Ashworth Scale; MA2, Melbourne Assessment 2; MEAN_SOL_STANCE, mean soleus (SOL) parameter during stance phase; PBS, Paediatric Balance Scale; PI, Pain Interference; PROM, passive range of motion; PROMIS-PI, Patient-Reported Outcomes Measurement Information System–Pain Interference; PT, physical therapy; QUEST, Quality of Upper Extremity Skills Test; RCT, randomised controlled trial; SCALE, Selective Control Assessment of the Lower Extremity; SCED, single-case experimental design; sdMoS, standard deviation of the margin of stability; SEAS, Self-reported Experiences of Activity Settings; VR, virtual reality.

**Table 2:** GRADE summary of findings for pre–post change after virtual reality-assisted rehabilitation in adolescents with cerebral palsy.

Outcome (Post-Intervention)	Importance	Studies (Participants)	Effect (Hedges’ g, 95% CI)	Heterogeneity (I^2^)	Certainty of Evidence (GRADE)	Comments
Lower-limb/balance-related function (various measures *)	Critical	4 (47)	0.21 (−0.17 to 0.58)	68%	Very low ⊕◯◯◯	a,b,c,d,e
Upper-limb function (various measures †)	Critical	3 (21)	0.47 (0.09 to 0.84)	72%	Very low ⊕◯◯◯	a,b,c,e

* Measures included PBS, GMFM-88, and gait speed; direction standardised so higher values indicate better function. † Measures included MA2, BBT, and QUEST (grasp domain); direction standardised so higher values indicate better function. a. Risk of bias (downgraded 2 levels, very serious): the evidence is primarily derived from uncontrolled pre–post designs (single-group pre–post and SCED). Even when a study was originally an RCT, only intervention-arm pre–post data were extracted for pooling to maintain consistency; therefore, confounding (natural recovery, regression to the mean, learning effects, co-interventions) cannot be ruled out. Most studies had unclear risk of bias, and one had high risk of bias. The feasibility of pooled between-group sensitivity analysis among controlled studies is summarised in Supplementary Table S10. b. Inconsistency (downgraded 1 level, serious): substantial heterogeneity was observed (I^2^ = 68% for lower-limb/balance; I^2^ = 72% for upper-limb), with few studies limiting exploration of sources. c. Imprecision (downgraded 1 level, serious): small total sample sizes (*n* = 47 and *n* = 21). For lower-limb/balance, the 95% CI crossed no effect (0); for upper-limb, the information size was very small despite CI not crossing 0. d. Indirectness (downgraded 1 level, serious; lower-limb/balance only): different instruments were used to represent the same functional domain (PBS, GMFM-88, gait speed), which may reflect partially different constructs within “lower-limb/balance-related function.” e. Publication bias/small-study effects: across all pooled studies (k = 7), possible small-study effects were suggested (Egger’s test *p* = 0.044; LFK index = 3.59), but interpretation was limited by the small number of studies and mixed outcome domains. GRADE symbols: ⊕ indicates a filled certainty symbol and ◯ indicates an empty certainty symbol; ⊕◯◯◯ represents very low certainty. **Abbreviations:** BBT, Box and Block Test; CI, confidence interval; GMFM-88, Gross Motor Function Measure-88; MA2, Melbourne Assessment 2; PBS, Paediatric Balance Scale; QUEST, Quality of Upper Extremity Skills Test; RCT, randomised controlled trial; SCED, single-case experimental design.

## Data Availability

The data supporting the findings of this review are available in the Supplementary Materials.

## Supplementary Materials

The following supporting information can be downloaded at: https://www.mdpi.com/article/10.3390/jcm15166248/s1, Supplementary Figure S1: Forest plot stratified by total intervention duration; Supplementary Figure S2: Forest plot stratified by device type; Supplementary Figure S3: Leave-one-out sensitivity analysis; Supplementary Figure S4: Funnel plot and small-study-effects diagnostics; Supplementary Table S1: Risk-of-bias/quality-assessment tool-specific results; Supplementary Table S2: Extracted outcome data for meta-analysis; Supplementary Table S3: Derivation/imputation log; Supplementary Table S4: GRADE evidence profile—pre–post change following virtual reality rehabilitation in adolescents with cerebral palsy; Supplementary Table S5: Outcome mapping and measurement instruments included in each pooled domain; Supplementary Table S6: Full-text reports excluded, with primary reasons; Supplementary Table S7: Full electronic search strategies; Supplementary Table S8: PRISMA 2020 checklist; Supplementary Table S9: Sensitivity analysis using alternative imputed within-participant pre–post correlation coefficients; Supplementary Table S10: Feasibility of between-group sensitivity analysis among controlled studies; Supplementary Table S11: Feasibility of additional heterogeneity and subgroup exploration; Supplementary Data S1: Meta-analysis dataset; Supplementary Data S2: Analysis settings log.
