# Supplementary material for: Virtual Reality-Assisted Rehabilitation for Adolescents with Cerebral Palsy: A Systematic Review and Meta-Analysis
Source: J Clin Med. 2026 Aug 12;15(16):6248. doi: 10.3390/jcm15166248 (PMC13513917; doi:10.3390/jcm15166248)
Supplement: Supplementary file 1 [file jcm-15-06248-s001.zip › Supplementary_Data_2_AnalysisSettingsLog_revised.pdf]

Analysis Settings Log

1. Data inputs

Primary dataset: Extracted outcome dataset (Table S2 / Supplementary Data 1). One effect size per study for the post-intervention endpoint (end of treatment).

Derivation / imputation log: Table S3 (units, directionality, subgroup reconstruction, SD handling, and assumed pre–post correlations).

2. Effect size definition

Effect measure: Hedges' g (standardized mean difference with small-sample correction).

Directionality: All outcomes were coded so that higher values indicate better function; therefore, positive Hedges' g values indicate improvement (see Table S3).

3. Primary meta-analysis (CMA)

Software: Comprehensive Meta-Analysis (CMA) v3.3.070 (Windows GUI).
Software add-on: MetaXL v5.3 (Excel add-in).
Date analyses last run: 2026-07-30.
Effect measure: Hedges' g (standardised mean difference with small-sample correction).
Model: Random-effects model was selected as the primary model for pooled effects.
Between-study variance (τ^2): estimated using the DerSimonian–Laird (DL) method within CMA v3.3.070.
Confidence interval (CI) / test method: CMA v3.3.070 z-based inference (normal approximation; two-tailed) was used for pooled estimates and hypothesis testing.
Number of studies: $k = 7$.

Primary pooled effects were obtained from the functional-domain subgroup analysis used to generate Figure 2. The prespecified functional-domain subgroups were lower-limb/balance-related function and upper-limb function. The overall pooled estimate was taken from the total row of the same subgroup-stratified CMA output.

Pooled effects from the functional-domain subgroup analysis (CMA):

Analysis level	Model	Number of studies	Hedges' g	SE	95% CI	Z	p
Lower-limb/balance-related function	Random effects	4	0.209	0.191	−0.167 to 0.584	1.090	0.276
Upper-limb function	Random effects	3	0.468	0.192	0.091 to 0.844	2.433	0.015
Overall, subgroup-stratified output	Random effects	7	0.338	0.136	0.072 to 0.603	2.489	0.013

Heterogeneity and subgroup difference:

Analysis level	Q	df	p	I^2 (%)	τ^2 (τ)
Lower-limb/balance-related function	9.272	3	0.026	67.644	0.099 (0.315)
Upper-limb function	7.048	2	0.029	71.625	0.062 (0.250)
Overall	21.695	6	0.001	72.344	0.075 (0.274)
Fixed-effect Q_between for subgroup difference	5.375	1	0.020	N/A	N/A

Reporting: random-effects estimates are reported as the primary pooled effects. The fixed-effect Q_between test from the CMA subgroup output was used to evaluate subgroup differences between prespecified functional domains. Because the number of studies was small and outcome measures were heterogeneous, subgroup comparisons were interpreted cautiously.

4. Publication bias / small-study effects

Methods performed in CMA:

Results are presented in Figure S4 (funnel plot and associated tests).

• Funnel plot (visual inspection).

• Egger's regression test for funnel plot asymmetry: $p = 0.044$.

• Trim-and-fill analysis: 0 studies imputed (no adjustment applied).

Additional method performed in MetaXL:

• IVhet model pooled estimate: Hedges' g = 0.215 (95% CI −0.072 to 0.502).

• Doi plot / Luis Furuya-Kanamori (LFK) index: 3.59 (major asymmetry; >2).

5. Additional analyses (as reported in Supplementary Figures)

Subgroup analyses:

• Device subgroup: commercial device vs rehabilitation-specific device.

• Duration subgroup: short-term (< 6 weeks) vs long-term (≥ 6 weeks), based on total intervention duration.

Sensitivity analyses:

• Leave-one-out influence analysis: see Figure S3.

Sensitivity analysis for imputed pre–post correlations:

Post hoc sensitivity analyses were performed to examine the robustness of the primary findings to the assumed within-participant pre–post correlation coefficient used for studies requiring imputed change-score standard deviations. Only studies without an empirically derived pre–post correlation were varied in this sensitivity analysis. Specifically, the imputed correlations for Jung et al. (2021) and Manikowska et al. (2021) were varied from $r = 0.50$ in the primary analysis to $r = 0.25$ and $r = 0.75$. Empirically derived correlations from adolescent participant-level or matched-pair data were retained for the remaining studies.

The interpretation of the findings did not materially change across the alternative r assumptions. Lower-limb/balance-related estimates remained statistically uncertain, upper-limb estimates remained positive, and overall pooled estimates remained positive. Detailed results are provided in Supplementary Table S9.

6. Key assumptions and reproducibility notes

• Pre–post correlations (r) for within-group pre–post effect size computation were specified per study in Table S2 and justified in Table S3.

• Any recalculation of adolescent-only (10–19 years) subgroup summary statistics and any reconstruction from stratified data are documented in Table S3.

• CMA is a GUI-based tool; therefore, this log records the software versions, key analysis choices (effect size metric, model type), and the observed outputs (pooled estimates and heterogeneity) to support reproducibility.
