# Supplementary material for: Virtual Reality-Assisted Rehabilitation for Adolescents with Cerebral Palsy: A Systematic Review and Meta-Analysis
Source: J Clin Med. 2026 Aug 12;15(16):6248. doi: 10.3390/jcm15166248 (PMC13513917; doi:10.3390/jcm15166248)
Supplement: Supplementary file 1 [file jcm-15-06248-s001.zip › Supplementary_Figure_S1_DurationSubgroup.pdf]

Study or Subgroup	Hedges' g	SE	Total	Weight	Hedges' g IV, Random, 95% CI
**Long-term (>= 6 weeks)**					
Jung et al. 2021	2.39	0.84	5	2.3%	〔0.75, 4.03〕
Meriggi et al. 2024	1.14	0.31	5	16.4%	〔0.53, 1.75〕
Hernandez et al. 2021	0.30	0.13	5	20.6%	〔0.04, 0.51〕
Manikowska et al. 2021	0.04	0.18	28	10.4%	〔−0.32, 0.40〕
Meyns et al. 2018	0.38	0.30	6	19.9%	〔−0.20, 0.97〕
Spomer et al. 2024	0.00	0.11	8	19.6%	〔−0.21, 0.21〕
**Subtotal(95% CI)**	**0.39**	**0.17**	**47**	**89.1%**	**〔0.04, 0.73〕**
Heterogeneity: $Tau^2 = 0.12$; $Chi^2 = 21.33$, df = 5 (P = 0.001) ; $I^2 = 77\%$					
Test for overall effect: Z = 2.21 (P = 0.03)					
**Short-term (< 6 weeks)**					
MacIntosh et al. 2020	0.28	0.12	11	10.9%	〔0.05, 0.54〕
**Subtotal(95% CI)**	**0.28**	**0.36**	**11**	**10.9%**	**〔−0.43, 0.99〕**
Heterogeneity: Not applicable					
Test for overall effect: Z = 0.77 (P = 0.44)					
**Total(95% CI)**	**0.37**	**0.16**	**68**	**100.0%**	**〔0.06, 0.67〕**
Heterogeneity: $Tau^2 = 0.08$; $Chi^2 = 21.70$, df = 6 (P = 0.001) ; $I^2 = 72\%$					
Test for overall effect: Z = 2.32 (P = 0.02)					
Test for subgroup differences: $Chi^2 = 0.36$, df = 1 (P = 0.55)					

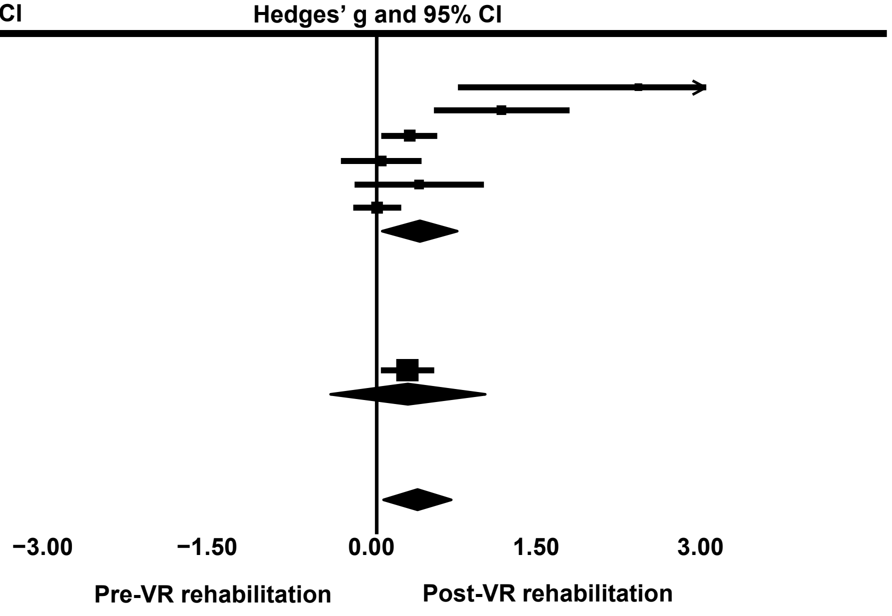

Supplementary Figure S1. Forest plot of within-group pre–post functional change after virtual reality–assisted rehabilitation, stratified by total intervention duration.

Effect sizes are presented as Hedges' g with 95% confidence intervals using random-effects models. Positive values indicate greater post-intervention improvement. Subgroup pooled estimates, the overall pooled estimate, and the test for subgroup differences are shown. Duration subgroup definitions were prespecified as short-term (<6 weeks) and long-term (≥6 weeks) according to total intervention duration.

Abbreviation: VR, virtual reality.
