# Supplementary material for: Virtual Reality-Assisted Rehabilitation for Adolescents with Cerebral Palsy: A Systematic Review and Meta-Analysis
Source: J Clin Med. 2026 Aug 12;15(16):6248. doi: 10.3390/jcm15166248 (PMC13513917; doi:10.3390/jcm15166248)
Supplement: Supplementary file 1 [file jcm-15-06248-s001.zip › Supplementary_Figure_S2_DeviceSubgroup.pdf]

Study or Subgroup	Hedges' g	SE	Total	Weight	Hedges' g IV, Random, 95% CI
**Commercial**					
Jung et al. 2021	2.39	0.84	5	2.3%	〔0.75, 4.03〕
Meyns et al. 2018	0.38	0.30	6	19.9%	〔−0.20, 0.97〕
**Subtotal(95% CI)**	**0.73**	**0.37**	**11**	**22.2%**	**〔0.01, 1.45〕**
Heterogeneity: $Tau^2 = 1.62$；$Chi^2 = 5.12$, df = 1 (P = 0.02)；$I^2 = 80\%$					
Test for overall effect: Z = 2.00 (P = 0.046)					
**Rehabilitation-specific**					
Meriggi et al. 2024	1.14	0.31	5	16.4%	〔0.53, 1.75〕
MacIntosh et al. 2020	0.28	0.12	11	10.9%	〔0.04, 0.51〕
Hernandez et al. 2021	0.30	0.13	5	20.6%	〔0.05, 0.54〕
Manikowska et al. 2021	0.04	0.18	28	10.4%	〔−0.32, 0.40〕
Spomer et al. 2024	0.00	0.11	8	19.6%	〔−0.21, 0.21〕
**Subtotal(95% CI)**	**0.27**	**0.14**	**57**	**77.8%**	**〔−0.01, 0.55〕**
Heterogeneity: $Tau^2 = 0.05$；$Chi^2 = 14.51$, df = 4 (P = 0.01)；$I^2 = 72\%$					
Test for overall effect: Z = 1.92 (P = 0.06)					
**Total(95% CI)**	**0.38**	**0.19**	**68**	**100.0%**	**〔0.002, 0.75〕**
Heterogeneity: $Tau^2 = 0.08$；$Chi^2 = 21.70$, df = 6 (P = 0.001)；$I^2 = 72\%$					
Test for overall effect: Z = 1.97 (P = 0.049)					
Test for subgroup differences: $Chi^2 = 2.06$, df = 1 (P = 0.15)					

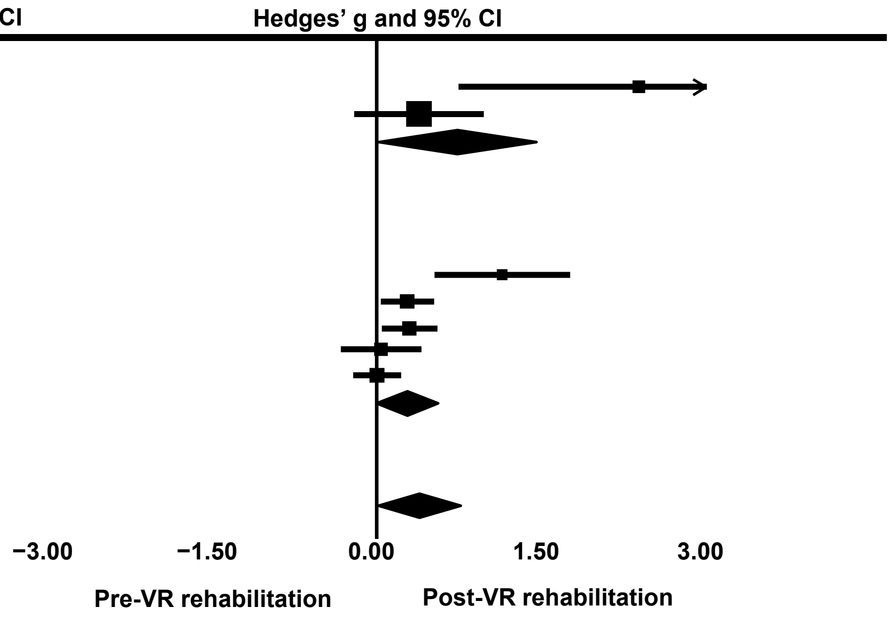

Supplementary Figure S2. Forest plot of within-group pre–post functional change after virtual reality–assisted rehabilitation, stratified by device type

Effect sizes are presented as Hedges' g with 95% confidence intervals using random-effects models. Positive values indicate greater post-intervention improvement. Subgroup pooled estimates, the overall pooled estimate, and the test for subgroup differences are shown. Device subgroups were defined as commercial devices (off-the-shelf consumer gaming or exergaming platforms) and rehabilitation-specific devices (purpose-built medical or rehabilitation VR systems described by the study authors).

Abbreviation: VR, virtual reality.
