# Supplementary material for: Virtual Reality-Assisted Rehabilitation for Adolescents with Cerebral Palsy: A Systematic Review and Meta-Analysis
Source: J Clin Med. 2026 Aug 12;15(16):6248. doi: 10.3390/jcm15166248 (PMC13513917; doi:10.3390/jcm15166248)
Supplement: Supplementary file 1 [file jcm-15-06248-s001.zip › Supplementary_Figure_S3_LeaveOneOut.pdf]

| Omitted study | Hedges' g | SE | Hedges' g Random, 95% CI | P-value |
|---|---|---|---|---|
| Jung et al. 2021 | 0.27 | 0.12 | 〔0.04, 0.50〕 | 0.019 |
| Meriggi et al. 2024 | 0.23 | 0.11 | 〔0.0004, 0.44〕 | 0.049 |
| MacIntosh et al. 2020 | 0.39 | 0.17 | 〔0.04, 0.73〕 | 0.027 |
| Hernandez et al. 2021 | 0.38 | 0.17 | 〔0.04, 0.71〕 | 0.028 |
| Manikowska et al. 2021 | 0.41 | 0.16 | 〔0.10, 0.71〕 | 0.009 |
| Meyns et al. 2018 | 0.34 | 0.15 | 〔0.05, 0.63〕 | 0.022 |
| Spomer et al. 2024 | 0.43 | 0.15 | 〔0.13, 0.73〕 | 0.006 |
| **Total(95% CI)** | **0.33** | **0.13** | **〔0.07, 0.60〕** | **0.012** |

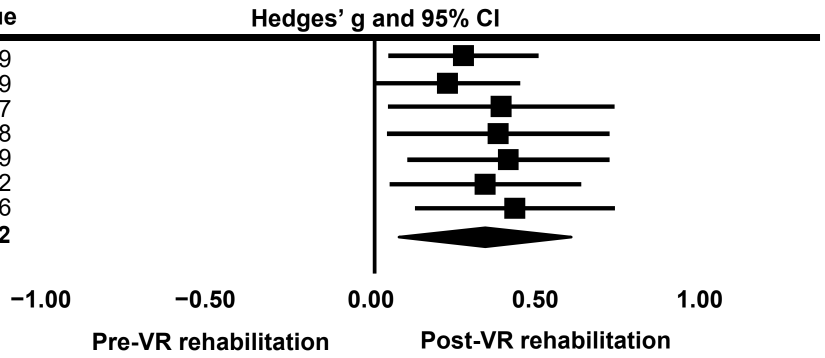


**Supplementary Figure S3.** Leave-one-out sensitivity analysis of the overall pooled effect

The pooled effect size was recalculated iteratively after omitting one study at a time to assess the influence of individual studies on the overall estimate. Effect sizes are presented as Hedges' g with 95% confidence intervals using random-effects models. Positive values indicate greater post-intervention improvement. The final row shows the overall pooled estimate including all studies.

**Abbreviation:** VR, virtual reality.
