# Supplementary material for: Virtual Reality-Assisted Rehabilitation for Adolescents with Cerebral Palsy: A Systematic Review and Meta-Analysis
Source: J Clin Med. 2026 Aug 12;15(16):6248. doi: 10.3390/jcm15166248 (PMC13513917; doi:10.3390/jcm15166248)
Supplement: Supplementary file 1 [file jcm-15-06248-s001.zip › Supplementary_Figure_S4_PublicationBias.pdf]

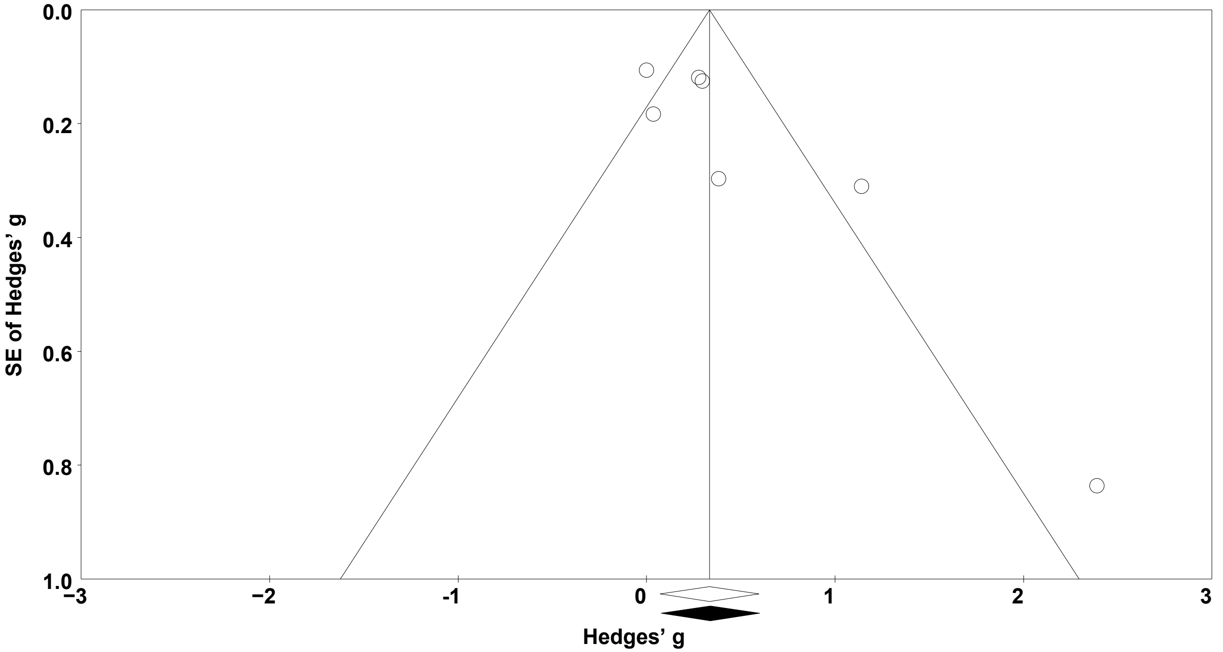

Supplementary Figure S4. Funnel plot and small-study-effects diagnostics for the overall pooled pre–post effect

The funnel plot shows within-group pre–post effect sizes (Hedges' g) plotted against their standard errors across pooled studies (k=7). Small-study effects were explored using Egger's regression test, trim-and-fill, and the Doi plot with the Luis Furuya-Kanamori (LFK) index. Egger's test suggested possible small-study effects (p=0.044), whereas trim-and-fill imputed no missing studies; the Doi plot showed major asymmetry (LFK index=3.59). Because the number of studies was small and diagnostics were applied to the overall pooled set spanning different outcome domains, findings should be interpreted cautiously.
