# Supplementary material for: Virtual Reality-Assisted Rehabilitation for Adolescents with Cerebral Palsy: A Systematic Review and Meta-Analysis
Source: J Clin Med. 2026 Aug 12;15(16):6248. doi: 10.3390/jcm15166248 (PMC13513917; doi:10.3390/jcm15166248)
Supplement: Supplementary file 1 [file jcm-15-06248-s001.zip › Supplementary_Table_S10_between_group_feasibility.pdf]

Supplementary Table S10. Feasibility of between-group sensitivity analysis among controlled studies.

Study	Design and participants	Intervention/comparator contrast	Controlled data available	Outcome domain and measures	Feasibility for between-group synthesis	Decision for this review
Jung et al. (2021)	Pilot randomized controlled trial; adolescents with spastic diplegia cerebral palsy, aged 11–17 years; KVG group n = 5 and conventional therapy group n = 5.	Both groups received conventional physical therapy. The KVG group received an additional 40 min of Xbox Kinect video game training per session, 3 sessions/week for 6 weeks; the comparator group received conventional therapy only.	Yes for the selected balance outcome. PBS pre/post and change values were reported for both arms: KVG 31.00 ± 6.20 to 47.80 ± 4.76, change 16.80 ± 1.44; control 33.40 ± 10.14 to 35.80 ± 10.13, change 2.40 ± 0.01. Between-group comparison favored KVG for PBS. SCALE was reported as median (range), and multiple gait variables were reported separately.	Lower-limb/balance-related function. Measures included SCALE, PBS, and GAITRite gait variables. PBS was the outcome used for the primary meta-analysis.	Not feasible for pooled between-group meta-analysis because this was the only randomized controlled trial contributing a compatible primary functional outcome. A pooled sensitivity analysis based on a single controlled contrast would not be statistically meaningful and would estimate a different contrast from the predominantly intervention-arm pre–post evidence base.	Narrative feasibility assessment only; not pooled. The controlled PBS result was considered when interpreting the lower-limb/balance findings and causal uncertainty.
Cardenas et al. (2021)	Case-comparison feasibility study with consecutive recruitment; children with cerebral palsy following lower extremity orthopedic surgery. The enrolled participants were within the adolescent age range used in this review: comparison group n = 5, age range 11–18 years; case group n = 5, age range 11–17 years.	The first five participants received standard physiotherapy (comparison group), and the next five received 15 cycling-based exergame sessions over 3 weeks in addition to standard physiotherapy (case group).	Yes for patient-reported secondary outcomes. Between-group Mann–Whitney U tests were reported for changes in FPS-R, PROMIS-PI, and KIDSCREEN-27 outcomes. No comparable lower-limb/balance or upper-limb functional outcome for the primary meta-analysis was reported.	Feasibility, pain, pain interference, health-related quality of life, well-being, target heart rate, and adverse events. Measures included FPS-R, PROMIS-PI, KIDSCREEN-27, gameplay heart-rate data, and interviews.	Not feasible for between-group functional synthesis because the outcomes were not compatible with the prespecified primary functional domains. In addition, the study was non-randomized and used a postoperative inpatient context, limiting comparability with the functional pre–post studies.	Narrative synthesis only. The study informed feasibility, pain, well-being, and safety-related interpretation rather than primary functional pooled estimates.

Note: The purpose of this table is to document whether a formal between-group sensitivity meta-analysis was feasible among studies with comparator groups. Randomized or non-randomized controlled contrasts estimate a different effect estimand from intervention-arm pre–post change; therefore, they were not pooled with the primary within-group synthesis.

A formal pooled between-group sensitivity meta-analysis was not performed because only one randomized controlled study provided a compatible primary functional outcome, while the other controlled study focused on feasibility, pain, well-being, and health-related quality-of-life outcomes rather than lower-limb/balance or upper-limb functional outcomes. Controlled findings were therefore incorporated narratively and used to qualify causal interpretation.

Abbreviations: CP, cerebral palsy; FPS-R, Faces Pain Scale-Revised; GAITRite, gait analysis system; KIDSCREEN-27, health-related quality-of-life questionnaire; KVG, Kinect video game; PBS, Pediatric Balance Scale; PROMIS-PI, Patient-Reported Outcome Measurement Information System Pediatric Pain Interference; SCALE, Selective Control Assessment of the Lower Extremity.
