# Supplementary material for: Virtual Reality-Assisted Rehabilitation for Adolescents with Cerebral Palsy: A Systematic Review and Meta-Analysis
Source: J Clin Med. 2026 Aug 12;15(16):6248. doi: 10.3390/jcm15166248 (PMC13513917; doi:10.3390/jcm15166248)
Supplement: Supplementary file 1 [file jcm-15-06248-s001.zip › Supplementary_Table_S11_heterogeneity_subgroup_feasibility.pdf]

Supplementary Table S11. Feasibility of additional heterogeneity and subgroup exploration.

This table summarises whether additional formal subgroup analyses could be performed for potential clinical, methodological, intervention, and outcome-related sources of heterogeneity beyond the prespecified functional-domain subgroup analysis and the previously reported duration/device subgroup analyses.

Potential heterogeneity source	Available information across included studies	Usable distribution for meta-analysis	Formal additional subgroup meta-analysis feasible?	Reason for feasibility decision	How addressed in the revised manuscript
Study design / comparator structure	Eight studies were included: five single-group pre-post studies, one RCT, one NRCT, and one SCED. Seven studies contributed to the within-group functional meta-analysis; Cardenas et al. was narratively synthesised.	Within-group synthesis: k = 7. Controlled functional contrast: only Jung et al. provided a compatible functional RCT outcome; Cardenas et al. focused on feasibility, pain, well-being, and heart-rate outcomes.	No	Combining within-group effects and between-group effects would mix different estimands. A pooled between-group sensitivity meta-analysis was not meaningful because only one RCT had a compatible primary functional outcome.	Added a controlled-study feasibility assessment in Supplementary Table S10 and clarified that causal inference is limited.
CP subtype	Reported subtypes included spastic bilateral, spastic unilateral, mixed, and not specified. Upper-limb studies mainly enrolled unilateral CP, whereas lower-limb/balance studies mainly enrolled bilateral CP or did not specify subtype.	Bilateral-dominant: Jung, Manikowska, Meyns. Unilateral-dominant: Meriggi, Hernandez. Mixed/unreported: MacIntosh, Spomer. These categories overlap strongly with outcome domain.	No	CP subtype is confounded with functional domain and intervention target. Subgroup cells would contain very few studies and would not isolate the independent effect of subtype.	Discussed CP subtype and intervention target as possible contributors to heterogeneity rather than performing a formal subgroup test.
Functional classification (GMFCS / MACS)	GMFCS was reported in several lower-limb/balance studies and ranged from levels I to IV. MACS was reported mainly in upper-limb studies and ranged from levels I to III. Several entries were unavailable or incompletely reported.	Reported classifications were not uniformly available across studies, and the relevant scale differed by intervention domain (GMFCS for gait/balance; MACS for upper limb).	No	The small number of studies and missing or non-comparable functional classification data precluded reliable subgroup pooling by GMFCS or MACS level.	Addressed as clinical heterogeneity and as a reason for cautious interpretation of pooled estimates.
VR technology / interface type	Platforms included Xbox Kinect systems, PC/Kinect, laptop plus Myo Armband, haptic device, robot-assisted gait/treadmill with VR feedback, stationary cycling plus PC exergames, and treadmill biofeedback.	Each technology/interface category contained one or very few studies and overlapped with outcome domain, setting, and intervention dose.	No	Too few studies per technology category. Interface type was highly confounded with the targeted body region and outcome measure.	Discussed platform specificity as a likely source of heterogeneity and retained the broader device subgroup analysis.
Commercial vs rehabilitation-specific device	Studies could be broadly grouped into commercially available exergaming systems versus rehabilitation-oriented or custom systems.	Commercial platforms: k = 2. Rehabilitation-specific/custom platforms: k = 5.	Already assessed	This was already examined as a prespecified/supplementary device subgroup analysis. No further subdivision was statistically reliable.	Reported in Additional Analyses and Supplementary Figure S2; interpreted cautiously due to small k and heterogeneity.
Intervention duration	Intervention duration varied from four total sessions to 12 weeks. Several studies delivered interventions at least 6 weeks, while one pooled study was shorter.	>=6 weeks: k = 6. <6 weeks: k = 1.	Already assessed	A duration subgroup was already reported; however, the <6-week subgroup contained one study, limiting inference.	Reported in Additional Analyses and Supplementary Figure S1; interpreted as exploratory.
Intervention dose / frequency / cumulative exposure	Frequency ranged from one to five sessions per week, session duration ranged from approximately 17 to 60 minutes, and total exposure varied substantially.	Dose metrics were not consistently reported in a format permitting comparable cumulative-dose calculation across all studies.	No	Heterogeneous and incompletely reported dose parameters prevented a reliable dose-response subgroup analysis or meta-regression.	Discussed dose and progression as possible clinical drivers and recommended transparent dose reporting in future trials.
Delivery setting and supervision	Studies included home-based interventions, clinical or rehabilitation-centre interventions, inpatient postoperative rehabilitation, and laboratory-based biofeedback/gait settings.	Setting categories were heterogeneous and often overlapped with device type and intervention target.	No	Too few studies per setting and major overlap with device, population, and outcome domain made a formal setting subgroup unreliable.	Addressed narratively as contextual heterogeneity relevant to implementation and generalisability.
Outcome domain	Primary pooled outcomes were prespecified as lower-limb/balance-related function and upper-limb function. Cardenas et al. provided patient-reported pain and well-being outcomes and was not pooled.	Lower-limb/balance: k = 4. Upper limb: k = 3.	Already assessed	This was the prespecified main subgroup structure of the primary forest plot.	Reported in the main Results section and Figure 2, with cautious interpretation due to very low certainty.
Outcome measure /	Lower-limb/balance outcomes included	Each instrument was generally	No	Instrument-level subgrouping would yield	Handled through domain mapping,

Potential heterogeneity source	Available information across included studies	Usable distribution for meta-analysis	Formal additional subgroup meta-analysis feasible?	Reason for feasibility decision	How addressed in the revised manuscript
measurement responsiveness	PBS, GMFM-88, and gait speed; upper-limb outcomes included MA2, BBT, and QUEST.	represented by one or very few studies.		single-study or very small subgroups and would not separate measurement responsiveness from intervention target.	GRADE indirectness for lower-limb/balance outcomes, and expanded discussion of measurement heterogeneity.
Age range / adolescent-specific extraction	Included studies either directly enrolled adolescents or had 10-19-year data extracted separately from mixed-age samples.	All included data met the adolescent eligibility criterion; age distributions remained narrow and study-level age ranges overlapped.	No	The review already restricted analyses to adolescent-specific data. Additional age subgroup analysis was not feasible with study-level aggregate data and small k.	Clarified adolescent-specific extraction criteria in Methods and retained age as part of the review eligibility framework.
Co-interventions and additional therapy exposure	Some studies combined VR-assisted rehabilitation with conventional physiotherapy or postoperative rehabilitation; co-intervention dose was not always equivalent between groups.	Co-intervention reporting and dose comparability were inconsistent.	No	Co-interventions differed across study designs and settings and could not be isolated statistically. In Jung et al., the VR group received additional training time compared with conventional therapy alone.	Discussed as a limitation of causal attribution and as a design issue for future controlled trials.
Adverse events / safety reporting	Adverse events were infrequently reported; no serious adverse events were described in the included literature.	Event data were too sparse and inconsistently reported for meta-analysis.	No	Inconsistent harms reporting and lack of event counts precluded quantitative safety synthesis.	Highlighted the need for standardised harms and tolerability reporting in future studies.

Note: Formal additional subgroup meta-analysis was considered inappropriate when subgroup categories contained very few studies, overlapped strongly with functional domain or intervention target, or lacked consistently reported data. These feasibility decisions were used to guide narrative interpretation rather than to generate additional underpowered subgroup estimates.

Abbreviations: BBT, Box and Block Test; CG, control group; CP, cerebral palsy; EMG, electromyography; GMFCS, Gross Motor Function Classification System; GMFM-88, Gross Motor Function Measure-88; IG, intervention group; IMU, inertial measurement unit; MACS, Manual Ability Classification System; MA2, Melbourne Assessment 2; NRCT, non-randomised controlled trial; PBS, Pediatric Balance Scale; QUEST, Quality of Upper Extremity Skills Test; RCT, randomised controlled trial; SCED, single-case experimental design; VR, virtual reality.
