# Supplementary material for: Virtual Reality-Assisted Rehabilitation for Adolescents with Cerebral Palsy: A Systematic Review and Meta-Analysis
Source: J Clin Med. 2026 Aug 12;15(16):6248. doi: 10.3390/jcm15166248 (PMC13513917; doi:10.3390/jcm15166248)
Supplement: Supplementary file 1 [file jcm-15-06248-s001.zip › Supplementary_Table_S1_RiskOfBias_ToolSpecific.pdf]

Supplementary Table S1a. NIH quality assessment (before–after studies) for studies included in the meta-analysis.

Study	Q1	Q2	Q3	Q4	Q5	Q6	Q7	Q8	Q9	Q10	Q11	Q12	Overall (Good/Fair/Poor)
Jung, S et al. (2021)	Yes	Yes	Yes	CD	No	Yes	Yes	Yes	Yes	Yes	No	NA	Fair
Meriggi, P et al. (2024)	Yes	Yes	Yes	CD	No	Yes	Yes	Yes	NR	Yes	No	NA	Poor
MacIntosh, A et al. (2020)	Yes	Yes	Yes	CD	Yes	Yes	Yes	No	Yes	No	No	NA	Fair
Hernandez, H et al. (2021)	Yes	Yes	Yes	CD	No	Yes	Yes	NR	Yes	Yes	No	NA	Fair
Manikowska, F et al. (2021)	Yes	Yes	Yes	CD	No	Yes	Yes	NR	Yes	Yes	No	NA	Fair
Meyns, P et al. (2018)	Yes	Yes	Yes	CD	No	Yes	Yes	NR	Yes	Yes	No	NA	Fair
Spomer, A. M. et al. (2024)	Yes	Yes	Yes	CD	No	Yes	Yes	NR	Yes	Yes	No	NA	Fair

Abbreviations: CD = cannot determine; NR = not reported; NA = not applicable. Overall rating follows NIH guidance (Good/Fair/Poor).

Supplementary Table S1b. SCED risk of bias assessment using RoBiNT.

Study	SCED design	I1	I2	I3	I4	I5	I6	I7	I8	I9	I10	I11	I12	I13	Overall (Low/Some concerns/High/Unclear)	Key limitations
MacIntosh, A et al. (2020)	Multiple baseline	Yes	Yes	Yes	Yes	Yes	Yes	Yes	Yes	No	Yes	Yes	Yes	Yes	Some concerns	Non-blinded design. Residual co-intervention confounding cannot be fully excluded.

Coding: Yes = low concern; No = high concern; NR = unclear/not reported.

Supplementary Table S1c. NRCT risk of bias assessment using ROBINS-I.

Study	Outcome/timepoint	D1 Confounding	D2 Selection	D3 Classification	D4 Deviations	D5 Missing	D6 Measurement	D7 Reporting	Overall	Key limitations
Cardenas, A et al. (2021)	KIDSCREEN-27 (HRQoL)/Post-intervention (end of program; after 3 weeks)	Serious	Serious	Moderate	Moderate	Low	Moderate	Moderate	Serious	Overall serious risk of bias driven by confounding and selection bias due to non-randomized sequential allocation and baseline imbalances between groups.

ROBINS-I domains: D1 = bias due to confounding; D2 = selection of participants; D3 = classification of interventions; D4 = deviations from intended interventions; D5 = missing data; D6 = measurement of outcomes; D7 = selection of the reported result. Judgments: Low/Moderate/Serious/Critical/No information.
