# Supplementary material for: Virtual Reality-Assisted Rehabilitation for Adolescents with Cerebral Palsy: A Systematic Review and Meta-Analysis
Source: J Clin Med. 2026 Aug 12;15(16):6248. doi: 10.3390/jcm15166248 (PMC13513917; doi:10.3390/jcm15166248)
Supplement: Supplementary file 1 [file jcm-15-06248-s001.zip › Supplementary_Table_S2_ExtractedOutcomeData.pdf]

Supplement Table S2. Extracted outcome data for meta-analysis

Study	Primary outcome	Timepoint (post)	n	Pre mean	Pre SD	Post mean	Post SD	Pre–post correlation (r) for CMA	Data handling
Jung et al. (2021)	PBS	End of week 6	5	31.00	6.20	47.80	4.76	0.5000	Direct extraction of group mean(SD) pre/post from main table; no individual participant data (IPD) available. Pre–post correlation set to r=0.50 (fixed) because r was not reported and additional r-based sensitivity would be arbitrary given aggregate-only reporting.
Meriggi et al. (2024)	MA2	End of week 10	5	64.82	4.82	76.35	7.98	0.8133	Recalculated adolescent-only (10–19y) subgroup mean(SD) from participant-level MA2 pre/post values reported in tables (IPD). Pearson r computed from matched pre/post pairs.
MacIntosh et al. (2020)	BBT	End of week 4	11	19.36	6.34	22.45	9.23	0.9118	Recalculated adolescent-only (10–19y) BBT mean(SD) from participant-level S/F values (IPD). Complete cases only (excluded participant(s) with missing post) to ensure consistent N. Pearson r computed from matched pre/post pairs.
Hernandez et al. (2021)	QUEST	End of week 12	5	58.58	19.30	68.92	11.28	0.9421	Recalculated adolescent-only (10–19y) QUEST domain mean(SD) from participant-level pre/post scores (IPD). Pearson r computed per domain from matched pre/post pairs.
Manikowska et al. (2021)	GMFM-88	End of week 8	28	50.56	23.71	51.44	23.03	0.5000	GMFM-88: overall mean reconstructed as weighted mean of GMFCS Group I/II means. SD not reported in-study; SD imputed using Rule B (external GMFM SDs stratified by GMFCS) and combined across strata; flagged for sensitivity analyses related to SD imputation. Pre–post correlation set to r=0.50 (fixed) because r was not reported; varying r would compound uncertainty on top of SD imputation.
Meyns et al. (2018)	PBS	End of week 6	6	48.50	6.03	51.00	3.9	0.6616	Recalculated adolescent-only (10–19y) PBS mean(SD) and Pearson r from participant-level pre/post PBS values (IPD). Also used as empirical PBS r source for r-imputation in PBS studies without r.
Spomer et al. (2024)	Gait speed	End of week 2	8	0.97	0.25	0.97	0.26	0.9423	Supporting information (CSV) provides de-identified IPD by limb. For speed, extracted affected limb only (SIDE == MORE_AFFECTED_LIMB) at pre/post, then recalculated mean(SD) and Pearson r from matched pre/post pairs.

Note: If a study reports multiple post-intervention timepoints, report the timepoint used in the meta-analysis and specify it in the Timepoint column.
