# Supplementary material for: Virtual Reality-Assisted Rehabilitation for Adolescents with Cerebral Palsy: A Systematic Review and Meta-Analysis
Source: J Clin Med. 2026 Aug 12;15(16):6248. doi: 10.3390/jcm15166248 (PMC13513917; doi:10.3390/jcm15166248)
Supplement: Supplementary file 1 [file jcm-15-06248-s001.zip › Supplementary_Table_S3_DerivationImputationLog_revised.pdf]

Supplement Table S3. Derivation / imputation log.

Study (Author Year)	Outcome (MA)	Timepoints	Adolescent handling (10–19y)	Data source (location)	Derivation / conversion (how obtained)	N used (pre/post)	Pre–post r (CMA)	Key assumptions / flags	Notes (for audit trail)
Jung et al. (2021)	PBS (Pediatric Balance Scale)	Baseline vs post (6 weeks)	All participants were adolescents (eligible: 10–19y)	Main text/table (group-level summary)	Direct extraction of group mean (SD) at pre and post. The pre–post correlation was not reported; r = 0.5000 was used in the primary CMA analysis, and alternative imputed r assumptions were assessed in sensitivity analysis.	5/5 per arm	0.5000	No IPD; aggregate-only reporting; pre–post r imputed for primary analysis.	Pilot RCT; r = 0.5000 used for the primary analysis because paired data were unavailable. Alternative assumptions of r = 0.25 and r = 0.75 were assessed in Supplementary Table S9.
Meriggi et al. (2024)	MA2 (Melbourne Assessment 2; Mean MA2 %)	T0 vs T1 (10 weeks)	Mixed ages; adolescent subgroup (10–19y) extracted; <10y excluded	Main tables with participant-level pre/post values (IPD)	Recalculated subgroup mean(SD) from adolescent IPD; Pearson r computed from matched pre/post pairs.	5/5	0.8133	Subgroup defined strictly by age	Domain: upper-limb function; values taken from Mean MA2 (%) per participant
MacIntosh et al. (2020)	BBT (Box and Blocks Test; blocks/min)	Starting (S) vs Finishing (F) (4 weeks)	Mixed ages; adolescent subgroup (10–19y) extracted; complete-case pairs only	Main table with participant-level S/F values (IPD)	Recalculated mean(SD) using adolescent complete cases; missing post excluded. Pearson r from matched pairs.	11/11	0.9118	One adolescent had missing post (“–”) → excluded for N consistency	Complete-case rule applied for pre/post and age summary to keep paired N
Hernandez et al. (2021)	QUEST (Quality of Upper Extremity Skills Test) — Grasp (%)	Pre vs post (12 weeks)	Mixed ages; adolescent subgroup (10–19y) extracted	Main tables with participant-level domain scores (IPD)	Recalculated subgroup mean(SD) from adolescent IPD. Pearson r computed for this domain from matched pairs. Age in years+months converted to decimal years (months/12) when needed.	5/5	0.9421	Domain-specific r; age conversion applied where applicable	Primary QUEST domain for MA: Grasp (activity-relevant hand function)
Manikowska et al. (2021)	GMFM-88 (Gross Motor Function Measure-88; total)	t1 (pre) vs t2 (post) (8 weeks)	All participants eligible (11.9–18.8y)	Main table reports GMFCS group means; SD not reported	Overall mean reconstructed as the weighted mean of GMFCS Group I/II means. SD imputed using Rule B (external GMFM SDs stratified by GMFCS strata) and combined across strata. The pre–post correlation was not reported; r = 0.5000 was used in the primary CMA analysis, and alternative imputed r assumptions were assessed in sensitivity analysis.	28/28	0.5000	SD imputed using external GMFM SDs; pre–post r imputed for primary analysis; included in leave-one-out and r-sensitivity analyses.	Flagged as an imputed-SD study in analyses and interpretation. Alternative assumptions of r = 0.25 and r = 0.75 were assessed in Supplementary Table S9.
Meyns et al. (2018)	PBS (Pediatric Balance Scale)	Pre-VGBT vs Post-VGBT (6 weeks)	Mixed ages; adolescent subgroup (10–19y) extracted; <10y excluded	Main table with participant-level PBS pre/post (IPD)	Recalculated mean(SD) from adolescent IPD; Pearson r computed from matched pre/post pairs.	6/6	0.6616	Provides empirical PBS r in adolescent CP	Used as the reference PBS r source when a PBS study lacks r
Spomer et al. (2024)	Raw gait speed (m/s; SPEED)	Pre vs post (2 weeks)	Adolescents with CP; all eligible	Supporting information (CSV; de-identified IPD by limb)	Affected limb only: selected rows where SIDE == MORE_AFFECTED_LIMB at each timepoint. Recalculated mean(SD) and Pearson r from matched pre/post pairs.	8/8	0.9423	Affected-limb-only rule applied prior to summary and r computation	Gait speed reported by limb; limb selection rule documented here

Directionality: All included outcomes were coded so that higher values indicate better function; accordingly, positive Hedges’ g values indicate improvement from pre- to post-intervention.

Abbreviations:

BBT, Box and Blocks Test; CMA, Comprehensive Meta-Analysis; CP, cerebral palsy; GMFCS, Gross Motor Function Classification System; GMFM-88, Gross Motor Function Measure-88; IPD, individual participant data; MA, meta-analysis; MA2, Melbourne Assessment 2; PBS, Pediatric Balance Scale; r, pre–post correlation (Pearson); SD, standard deviation; VGT, video game training; VR, virtual reality.
