# Supplementary material for: Virtual Reality-Assisted Rehabilitation for Adolescents with Cerebral Palsy: A Systematic Review and Meta-Analysis
Source: J Clin Med. 2026 Aug 12;15(16):6248. doi: 10.3390/jcm15166248 (PMC13513917; doi:10.3390/jcm15166248)
Supplement: Supplementary file 1 [file jcm-15-06248-s001.zip › Supplementary_Table_S4_GRADE_EvidenceProfile.pdf]

Table S4. GRADE evidence profile — Pre–post change following VR rehabilitation in adolescents with cerebral palsy (10–19 years)

Outcome	Studies (participants)	Study design contributing to pooled estimate	Risk of bias	Inconsistency	Indirectness	Imprecision	Publication bias	Overall certainty
Lower-limb/balance-related function	4 (47)	Predominantly uncontrolled pre–post (single-group pre–post); one study originally RCT but pooled using intervention-arm pre–post only	Very serious (−2)*	Serious (−1)<br>I^2=68%	Serious (−1)<br>Mixed constructs/instruments	Serious (−1)<br>CI crosses 0, small n	Suspected (0)†	Very low
Upper-limb function	3 (21)	Uncontrolled pre–post (single-group pre–post) and SCED	Very serious (−2)*	Serious (−1)<br>I^2=72%	Not serious (0)	Serious (−1)<br>Very small n	Suspected (0)†	Very low

* Risk of bias rationale: evidence primarily from uncontrolled pre–post/SCED; confounding cannot be ruled out; most studies rated unclear RoB and one high RoB.

† Overall k=7: funnel asymmetry; Egger p=0.044; LFK=3.59; trim-and-fill 0; limited by small k and mixed domains
