# Supplementary material for: Virtual Reality-Assisted Rehabilitation for Adolescents with Cerebral Palsy: A Systematic Review and Meta-Analysis
Source: J Clin Med. 2026 Aug 12;15(16):6248. doi: 10.3390/jcm15166248 (PMC13513917; doi:10.3390/jcm15166248)
Supplement: Supplementary file 1 [file jcm-15-06248-s001.zip › Supplementary_Table_S5_OutcomeMapping_Instruments.pdf]

Table S5. Outcome mapping and measurement instruments included in each pooled domain.

Study	Domain subgroup	Instrument used for effect size	Construct label	Direction standardized (higher=better)	Notes
Jung, S et al. (2021)	Lower limb/balance	PBS	Balance	Yes	Included in pooled lower-limb/balance
Meriggi, P et al. (2024)	Upper limb	MA2	Upper limb function/movement quality	Yes	Included in pooled upper-limb
MacIntosh, A et al. (2020)	Upper limb	BBT	Manual dexterity	Yes	Included in pooled upper-limb
Hernandez, H et al. (2021)	Upper limb	QUEST (grasp)	Upper limb function (grasp)	Yes	Included in pooled upper-limb
Manikowska, F et al. (2021)	Lower limb/balance	GMFM-88	Gross motor function/mobility	Yes	Included in pooled lower-limb/balance
Meyns, P et al. (2018)	Lower limb/balance	PBS	Balance	Yes	Included in pooled lower-limb/balance
Spomer, A. M. et al. (2024)	Lower limb/balance	Gait speed	Walking/mobility	Yes	Included in pooled lower-limb/balance

Note: For consistency across study designs, when a study was originally an RCT, only intervention-arm pre–post data were extracted for pooling.
