# Supplementary material for: Virtual Reality-Assisted Rehabilitation for Adolescents with Cerebral Palsy: A Systematic Review and Meta-Analysis
Source: J Clin Med. 2026 Aug 12;15(16):6248. doi: 10.3390/jcm15166248 (PMC13513917; doi:10.3390/jcm15166248)
Supplement: Supplementary file 1 [file jcm-15-06248-s001.zip › Supplementary_Table_S6_ExcludedFullText.pdf]

Report / Record (Year)	Title / Identifier	Source	Primary reason	Notes
Barton (2013)	The effects of virtual reality game…	Journal of Neuroengineering and Rehabilitation	Ineligible study design	Case report
Brien (2011)	An intensive virtual reality program…	Pediatric Physical Therapy	Ineligible study design	Case series with n < 5
Camardella (2023)	Does the Level of Focus in Serious…	IEEE International Conference on Rehabilitation Robotics	Ineligible study design	Case series with adolescent population n < 5
Chan-Viquez (2023)	Understanding a videogame home…	Frontiers in Medical Technology	Ineligible study design	Case series with n < 5
Chen (2015)	Effect of a Home-Based Virtual…	Rehabilitation Research & Practice	Ineligible study design	Case series with n < 5
Eckert (2023)	Finding Effective Adjustment Levels…	JMIR Serious Games	Ineligible study design	Case series with adolescent population n < 5
Gehringer (2024)	Feasibility of At-Home Hand Arm…	JMIR Formative Research	Ineligible study design	Case report
Gonzalez (2010)	Vision based interface: an alternative…	Annual International Conference Of The IEEE Engineering In Medicine And Biology Society	Ineligible study design	Case series with n < 5
Hung (2018)	Virtual reality training for children…	Developmental Medicine and Child Neurology	Ineligible study design	Commentary article
Jung (2018)	Does virtual reality training using…	Journal of Pediatric Rehabilitation Medicine	Ineligible study design	Case series with n < 5
Kassee (2017)	Home-based Nintendo Wii training…	Journal of Pediatric Rehabilitation Medicine	Ineligible study design	Case series with adolescent population n < 5
Lee (2016)	Using health games for rehabilitation…	Journal of Physical Therapy Science	Ineligible study design	Case series with adolescent population n < 5
Levin (2017)	Gait Training With Visual Feedback…	Pediatric Physical Therapy	Ineligible study design	Case series with adolescent population n < 5
Lin (2015)	Interactive augmented reality using…	Research in Developmental Disabilities	Ineligible study design	Case series with n < 5
Menici (2022)	Virtual-rehabilitation for postural…	Developmental Medicine and Child Neurology	Ineligible study design	Case report
Patritti (2010)	The role of augmented feedback in…	Technology and Disability	Ineligible study design	Case series with n < 5
Peper (2013)	Bimanual training for children with…	Developmental Neurorehabilitation	Ineligible study design	Case series with adolescent population n < 5
Reid (2002)	The use of virtual reality to improve…	Technology and Disability	Ineligible study design	Case series with n < 5
Rios (2013)	NeuroGame Therapy to improve…	Developmental Neurorehabilitation	Ineligible study design	Case series with n < 5
Shields (2015)	A web-based therapy program…	Journal of Physiotherapy	Ineligible study design	Critical appraisal of an RCT
Takashio (2012)	Using a virtual reality-based therapy…	Developmental Medicine and Child Neurology	Ineligible study design	Case series with adolescent population n < 5
Tsai (2011)	Changes in anticipatory postural…	Physiotherapy (United Kingdom)	Ineligible study design	Case series with adolescent population n < 5
Valenzuela (2021)	Intensive Training with Virtual…	International Journal of Environmental Research & Public Health	Ineligible study design	Case series with n < 5
Vergara-Diaz (2017)	Cervical posture therapy using a…	Archives of Physical Medicine and Rehabilitation	Ineligible study design	Case series with n < 5
Winkels (2013)	Wii TM-habilitation of upper…	Developmental Neurorehabilitation	Ineligible study design	Case series with adolescent population n < 5

Wood (2013)	Feasibility of gestural feedback…	IEEE Transactions on Neural Systems and Rehabilitation Engineering	Ineligible study design	Case series with adolescent population n < 5
Wu (2011)	Combined passive stretching and…	Neurorehabilitation and Neural Repair	Ineligible study design	Case series with adolescent population n < 5
Lai (2025)	School-Based Virtual Reality…	JMIR Formative Research	Ineligible population	Mixed diagnoses (Autism, ADHD, etc.)
Lopez-Ortiz (2013)	Motor learning in virtual reality as a…	Developmental Medicine and Child Neurology	Ineligible intervention	Intervention not aligned with VR-assisted rehabilitation (VR task used as assessment/marker)
Metin Ökmen (2013)	The effect of virtual reality therapy…	Noropsikiyatri Arsivi	Ineligible outcomes	Psychological adaptation only; no prespecified functional outcomes reported
Robert (2018)	Validation of reaching in a virtual…	Developmental Medicine and Child Neurology	Ineligible intervention	VR used for assessment/validation, not VR-assisted rehabilitation
Shin (2015)	Effects of conventional neurological…	Journal of Physical Therapy Science	Ineligible population	No adolescents 10–19
Wick (2010)	Virtual-reality-based Paediatric…	Developmental Medicine and Child Neurology	Ineligible population	Age not reported; unable to confirm 10–19-year eligibility
ACTRN12611001174976 (2011)	MiTii: a randomised trial of novel…	ACTRN registry	Protocol only / ongoing trial	No posted results
ACTRN12619000781145 (2019)	The effect of a virtual reality game…	ACTRN registry	Protocol only / ongoing trial	No posted results
ACTRN12622001013752 (2022)	The Effects of Virtual Reality-Based…	ACTRN registry	Protocol only / ongoing trial	No posted results
ChiCTR2100044090 (2021)	Study on the rehabilitation effect of…	ChiCTR registry	Protocol only / ongoing trial	No posted results
ChiCTR2300069853 (2023)	Effect of virtual reality combined…	ChiCTR registry	Protocol only / ongoing trial	No posted results
ChiCTR2300074122 (2023)	Effect of virtual reality on upper…	ChiCTR registry	Protocol only / ongoing trial	No posted results
CTRI/2011/11/002137 (2011)	Use of Nintendo Wii, a commercial…	CTRI registry	Protocol only / ongoing trial	No posted results
CTRI/2017/12/011045 (2017)	Effects of Microsoft kinetic…	CTRI registry	Protocol only / ongoing trial	No posted results
CTRI/2024/02/062656 (2024)	Effectiveness of Virtual reality on…	CTRI registry	Protocol only / ongoing trial	No posted results
CTRI/2024/07/071345 (2024)	Enhancing Balance in Children with…	CTRI registry	Protocol only / ongoing trial	No posted results
CTRI/2024/03/063762 (2024)	Virtual Reality and Balance: helping…	CTRI registry	Protocol only / ongoing trial	No posted results
CTRI/2025/05/086304 (2025)	Effectiveness of Virtual Reality…	CTRI registry	Protocol only / ongoing trial	No posted results
CTRI/2025/07/091640 (2025)	How Game based Rehab Helps…	CTRI registry	Protocol only / ongoing trial	No posted results
CTRI/2025/06/088889 (2025)	Improving Trunk Control and…	CTRI registry	Protocol only / ongoing trial	No posted results
DRKS00012690 (2018)	Therapy accompanying use of VR…	DRKS registry	Protocol only / ongoing trial	No posted results

Gatica-Rojas (2017)	Effectiveness of a Nintendo Wii…	Contemporary Clinical Trials Communications	Protocol only / ongoing trial	No posted results
Gatica-Rojas (2023)	Postural control telerehabilitation…	PLoS ONE [Electronic Resource]	Protocol only / ongoing trial	No posted results
Guinet (2022)	Effect of an augmented reality active…	BMJ Open	Protocol only / ongoing trial	No posted results
Guinet (2022)	ARRoW-CP: Gait rehabilitation…	Developmental Medicine and Child Neurology	Protocol only / ongoing trial	No posted results
Guinet (2020)	Exploring visual feedback modalities…	Gait and Posture	Protocol only / ongoing trial	No posted results
Iqbal (2024)	Evaluating the effectiveness of…	MethodsX	Protocol only / ongoing trial	No posted results
IRCT201010164945N1 (2011)	Efficacy comparison between virtual…	IRCT registry	Protocol only / ongoing trial	No posted results
IRCT201101244945N2 (2011)	Effects of Sensory Motor Plays in…	IRCT registry	Protocol only / ongoing trial	No posted results
IRCT20090301001722N25 (2020)	Effectiveness of virtual reality…	IRCT registry	Protocol only / ongoing trial	No posted results
IRCT20200415047094N1 (2020)	The Effect of Virtual Reality on…	IRCT registry	Protocol only / ongoing trial	No posted results
IRCT20211024052860N1 (2022)	The effect of virtual reality camera…	IRCT registry	Protocol only / ongoing trial	No posted results
IRCT20220627055296N1 (2022)	Effect of virtual reality on hand…	IRCT registry	Protocol only / ongoing trial	No posted results
IRCT20230907059375N1 (2024)	Comparsion of virtual reality task…	IRCT registry	Protocol only / ongoing trial	No posted results
IRCT20241001063235N1 (2024)	Effect of virtual reality in children…	IRCT registry	Protocol only / ongoing trial	No posted results
ISRCTN91944190 (2012)	Limbs Alive: use of video games to…	ISRCTN registry	Protocol only / ongoing trial	No posted results
ISRCTN26206379 (2013)	To evaluate increased benefits to…	ISRCTN registry	Protocol only / ongoing trial	No posted results
ISRCTN80878394 (2021)	Does a 10-week physiotherapy…	ISRCTN registry	Protocol only / ongoing trial	No posted results
Kanitkar (2017)	The Effectiveness of a Computer…	JMIR Research Protocols	Protocol only / ongoing trial	No posted results
Kazemnazhand (2025)	Impact of computerised visual-motor…	BMJ Open	Protocol only / ongoing trial	No posted results
KCT0002395 (2017)	The effect of digital rehabilitation…	KCT registry	Protocol only / ongoing trial	No posted results
KCT0010888 (2025)	Effects of an immersive virtual…	KCT registry	Protocol only / ongoing trial	No posted results
Lai (2022)	The Effects of Virtual Reality…	JMIR Research Protocols	Protocol only / ongoing trial	No posted results
Li (2023)	Effect of virtual reality combined…	BMC Neurology	Protocol only / ongoing trial	No posted results
McNish (2019)	Rehabilitation for Children With…	JMIR Research Protocols	Protocol only / ongoing trial	No posted results
NCT01700153 (2012)	Efficacy Study of an Interactive…	NCT registry	Protocol only / ongoing trial	No posted results
NCT02039284 (2013)	Efficacy of New Protocols in the…	NCT registry	Protocol only / ongoing trial	No posted results
NCT01901211 (2013)	Physical and Social Benefits of…	NCT registry	Protocol only / ongoing trial	No posted results
NCT02290353 (2014)	Functional Engagement in Assisted…	NCT registry	Protocol only / ongoing trial	No posted results

NCT02364245 (2015)	Evaluation the Feasibility and…	NCT registry	Protocol only / ongoing trial	No posted results
NCT04483388 (2015)	Training With Virtual Reality in…	NCT registry	Protocol only / ongoing trial	No posted results
NCT02949817 (2016)	The Effect of Digital Rehabilitation…	NCT registry	Protocol only / ongoing trial	No posted results
NCT02841319 (2016)	The Effects of Virtual Reality on…	NCT registry	Protocol only / ongoing trial	No posted results
NCT02728375 (2016)	Game-Based Rehabilitation Platform…	NCT registry	Protocol only / ongoing trial	No posted results
NCT02975804 (2016)	RCT on Interactive Computer Play…	NCT registry	Protocol only / ongoing trial	No posted results
NCT02938754 (2016)	Rehabilitation Gaming System for…	NCT registry	Protocol only / ongoing trial	No posted results
NCT03353623 (2017)	Immersive Virtual Environments and…	NCT registry	Protocol only / ongoing trial	No posted results
NCT03078998 (2017)	Leap Motion Based Exercises in…	NCT registry	Protocol only / ongoing trial	No posted results
NCT03386968 (2017)	Virtual Reality and Active Video…	NCT registry	Protocol only / ongoing trial	No posted results
NCT03700073 (2018)	Correlation Physiological Cost Index…	NCT registry	Protocol only / ongoing trial	No posted results
NCT03622970 (2018)	Video-Based Games for Upper Limb…	NCT registry	Protocol only / ongoing trial	No posted results
NCT03889262 (2019)	Development of Simulated…	NCT registry	Protocol only / ongoing trial	No posted results
NCT04147468 (2019)	Functional Strength Training and…	NCT registry	Protocol only / ongoing trial	No posted results
NCT03811275 (2019)	Task or Virtual Reality Intervention…	NCT registry	Protocol only / ongoing trial	No posted results
NCT04630678 (2020)	Individualized Virtual Reality in the…	NCT registry	Protocol only / ongoing trial	No posted results
NCT04679779 (2020)	Virtual Reality in Children With…	NCT registry	Protocol only / ongoing trial	No posted results
NCT04529343 (2020)	Virtual Reality Mediated Upper…	NCT registry	Protocol only / ongoing trial	No posted results
NCT04467385 (2020)	Virtual Reality Training With…	NCT registry	Protocol only / ongoing trial	No posted results
NCT04802655 (2021)	Effect of Exergame Program…	NCT registry	Protocol only / ongoing trial	No posted results
NCT04768374 (2021)	Effects of Virtual Reality on…	NCT registry	Protocol only / ongoing trial	No posted results
NCT04954144 (2021)	Role of Virtual Reality in Hand…	NCT registry	Protocol only / ongoing trial	No posted results
NCT05557305 (2022)	Changes in Motor and Cognitive…	NCT registry	Protocol only / ongoing trial	No posted results
NCT05327179 (2022)	Effects of Action Observation…	NCT registry	Protocol only / ongoing trial	No posted results
NCT05411328 (2022)	Efficacy of Virtual Reality on…	NCT registry	Protocol only / ongoing trial	No posted results
NCT05667792 (2022)	Examination of the Effects of…	NCT registry	Protocol only / ongoing trial	No posted results
NCT05299905 (2022)	ImGTS for Patients With Cerebral…	NCT registry	Protocol only / ongoing trial	No posted results
NCT05670522 (2022)	Transcranial Direct Current…	NCT registry	Protocol only / ongoing trial	No posted results
NCT05368922 (2022)	Upper Limb Rehabilitation Using…	NCT registry	Protocol only / ongoing trial	No posted results

NCT05463718 (2022)	Virtual Reality Versus Balance Beam…	NCT registry	Protocol only / ongoing trial	No posted results
NCT05494905 (2022)	Virtual Reality vs Functional…	NCT registry	Protocol only / ongoing trial	No posted results
NCT05740020 (2023)	Effect of Virtual Reality in Spider…	NCT registry	Protocol only / ongoing trial	No posted results
NCT06240988 (2023)	Effects of CIMT With and Without…	NCT registry	Protocol only / ongoing trial	No posted results
NCT05931640 (2023)	Effects of Plyometric Training…	NCT registry	Protocol only / ongoing trial	No posted results
NCT05988944 (2023)	The Hand Functions In Cerebral…	NCT registry	Protocol only / ongoing trial	No posted results
NCT06100341 (2023)	ImGTS for Patients With Cerebral…	NCT registry	Protocol only / ongoing trial	No posted results
NCT06214364 (2023)	Transcranial Direct Current…	NCT registry	Protocol only / ongoing trial	No posted results
NCT06073743 (2023)	Video Based Games Exercise…	NCT registry	Protocol only / ongoing trial	No posted results
NCT05846542 (2023)	Video-game Based Therapy in…	NCT registry	Protocol only / ongoing trial	No posted results
NCT05872217 (2023)	Virtual reality technology…	NCT registry	Protocol only / ongoing trial	No posted results
NCT06506682 (2024)	Comparing CIMT With Virtual…	NCT registry	Protocol only / ongoing trial	No posted results
NCT06571929 (2024)	Effect of Task-Specific Training in…	NCT registry	Protocol only / ongoing trial	No posted results
NCT06317935 (2024)	The Effect of Virtual Reality…	NCT registry	Protocol only / ongoing trial	No posted results
NCT06520644 (2024)	Effect Of Virtual Reality On…	NCT registry	Protocol only / ongoing trial	No posted results
NCT06398119 (2024)	Effect of Visuomotor Training Using…	NCT registry	Protocol only / ongoing trial	No posted results
NCT06303440 (2024)	Effects of Virtual Reality Versus…	NCT registry	Protocol only / ongoing trial	No posted results
NCT06428292 (2024)	Fully Immersive Virtual Reality…	NCT registry	Protocol only / ongoing trial	No posted results
NCT07041944 (2025)	HEMImprove 1.0 in Children and…	NCT registry	Protocol only / ongoing trial	No posted results
NCT06821815 (2025)	Intensive Video-Based Game…	NCT registry	Protocol only / ongoing trial	No posted results
NCT06846190 (2025)	Modified Constraint Induced…	NCT registry	Protocol only / ongoing trial	No posted results
NCT07063810 (2025)	NEO-REEDUC - Mixed Reality for…	NCT registry	Protocol only / ongoing trial	No posted results
NCT06817941 (2025)	Speech and Arm Combined…	NCT registry	Protocol only / ongoing trial	No posted results
PACTR202502864625688 (2025)	Effect of Gamified Balance Training…	PACTR registry	Protocol only / ongoing trial	No posted results
Petrevska (2024)	Evaluating the impact of movement…	BMJ Open	Protocol only / ongoing trial	No posted results
RBR-3sc9zc (2015)	Impact of training with video games…	RBR registry	Protocol only / ongoing trial	No posted results
Tarakci (2018)	Investigation of the effectiveness of…	Developmental Medicine and Child Neurology	Protocol only / ongoing trial	No posted results
TCTR20200817001 (2020)	Effect of Motor Sensing Input-based…	TCTR registry	Protocol only / ongoing trial	No posted results
RBR-3zty4w (2018)	Effects of active videogame system…	RBR registry	Protocol only / ongoing trial	No posted results

RBR-6zyrzjq (2025)	The Effects of Virtual Reality…	RBR registry	Protocol only / ongoing trial	No posted results
Kim (2019)	Effects of Virtual Reality Horse…	The Journal of Korean Physical Therapy	Insufficient data for extraction	10–19 y subgroup outcomes not reported
Acar (2016)	Efficacy of neurodevelopmental…	Journal of Physical Therapy Science	Insufficient data for extraction	10–19 y subgroup outcomes not reported
Adamovich (2010)	Robotically facilitated upper…	Archives of Physical Medicine and Rehabilitation	Insufficient data for extraction	10–19 y subgroup outcomes not reported
Almuwais (2015)	The feasibility and effectiveness of…	Physiotherapy (United Kingdom)	Insufficient data for extraction	10–19 y subgroup outcomes not reported
AlSaif (2015)	Effects of interactive games on…	Journal of Physical Therapy Science	Insufficient data for extraction	10–19 y subgroup outcomes not reported
Arnoni (2019)	Effects of virtual reality in body…	Complementary Therapies in Clinical Practice	Insufficient data for extraction	10–19 y subgroup outcomes not reported
Ashrafova (2024)	Possibility of using the C-Mill…	BMJ Paediatrics Open	Insufficient data for extraction	10–19 y subgroup outcomes not reported
Ata (2020)	Effect of Virtual Reality on Balance…	Archives of Physical Medicine and Rehabilitation	Insufficient data for extraction	10–19 y subgroup outcomes not reported
Ata (2020)	Effect of Xbox Kinect on Balance in…	Archives of Physical Medicine and Rehabilitation	Insufficient data for extraction	10–19 y subgroup outcomes not reported
Ata (2020)	Effect of Xbox kinect on balance in…	Developmental Medicine and Child Neurology	Insufficient data for extraction	10–19 y subgroup outcomes not reported
Ballaz (2014)	Impaired visually guided weight…	Research in Developmental Disabilities	Insufficient data for extraction	10–19 y subgroup outcomes not reported
Bedair (2016)	Impact of virtual reality games as an…	International Journal of PharmTech Research	Insufficient data for extraction	10–19 y subgroup outcomes not reported
Bilir (2025)	The impact of motion capture…	Turkish Journal of Physical Medicine and Rehabilitation	Insufficient data for extraction	10–19 y subgroup outcomes not reported
Bonello (2024)	Unveiling the impact of the…	Computational And Structural Biotechnology Journal	Insufficient data for extraction	10–19 y subgroup outcomes not reported
Bonnechere (2017)	Balance improvement after physical…	Disability and Rehabilitation	Insufficient data for extraction	10–19 y subgroup outcomes not reported
Bortone (2020)	Immersive Virtual Environments and…	Journal of Neuroengineering and Rehabilitation	Insufficient data for extraction	10–19 y subgroup outcomes not reported
Bortone (2018)	Wearable Haptics and Immersive…	IEEE Transactions on Neural Systems and Rehabilitation Engineering	Insufficient data for extraction	10–19 y subgroup outcomes not reported
Boulay (2022)	Effectiveness of serious games in…	Gait and Posture	Insufficient data for extraction	10–19 y subgroup outcomes not reported
Camara (2017)	Motor Improvement Using Motion…	Journal of Motor Behavior	Insufficient data for extraction	10–19 y subgroup outcomes not reported
Camardella (2025)	The Wizard Apprentice: A Serious…	IEEE Transactions on Neural Systems and Rehabilitation Engineering	Insufficient data for extraction	10–19 y subgroup outcomes not reported
Chang (2021)	Virtual Reality-Incorporated Horse…	Sensors	Insufficient data for extraction	10–19 y subgroup outcomes not reported
Chang (2020)	Effects of Virtual Reality-Based…	Healthcare	Insufficient data for extraction	10–19 y subgroup outcomes not reported
Chan-Viquez (2024)	Feasibility of a home-based home…	Journal of Neuroengineering and Rehabilitation	Insufficient data for extraction	10–19 y subgroup outcomes not reported
Chen (2012)	Muscle strength enhancement…	Research in Developmental Disabilities	Insufficient data for extraction	10–19 y subgroup outcomes not reported
Chen (2021)	Development and Feasibility of a…	Frontiers in Bioengineering & Biotechnology	Insufficient data for extraction	10–19 y subgroup outcomes not reported
Chen (2014)	Home-based tele-assisted robotic…	Annual International Conference Of The IEEE Engineering In	Insufficient data for extraction	10–19 y subgroup outcomes not reported

		Medicine And Biology Society		
Chen (2007)	Use of virtual reality to improve…	Physical Therapy	Insufficient data for extraction	10–19 y subgroup outcomes not reported
Cho (2016)	Treadmill Training with Virtual…	Tohoku Journal of Experimental Medicine	Insufficient data for extraction	10–19 y subgroup outcomes not reported
Choi (2021)	Virtual reality rehabilitation in…	Developmental Medicine and Child Neurology	Insufficient data for extraction	10–19 y subgroup outcomes not reported
Choi (2023)	Home-based virtual reality-enhanced…	Frontiers in Pediatrics	Insufficient data for extraction	10–19 y subgroup outcomes not reported
Coley (2022)	PedBotHome: A Video Game-Based…	Pediatric Physical Therapy	Insufficient data for extraction	10–19 y subgroup outcomes not reported
Curtis (2014)	The effect of training in an…	Developmental Neurorehabilitation	Insufficient data for extraction	10–19 y subgroup outcomes not reported
Daliri (2023)	Investigating the Effect of Leap…	Developmental Neurorehabilitation	Insufficient data for extraction	10–19 y subgroup outcomes not reported
Daliri (2025)	Impact of Virtual Reality Task…	Pediatric Neurology	Insufficient data for extraction	10–19 y subgroup outcomes not reported
Decavele (2019)	A randomized cross-over design…	Developmental Medicine and Child Neurology	Insufficient data for extraction	10–19 y subgroup outcomes not reported
Delgado (2016)	Augmenting constraint induced…	Developmental Medicine and Child Neurology	Insufficient data for extraction	10–19 y subgroup outcomes not reported
El-Shamy (2020)	Effect of Wii training on hand…	Physiotherapy Theory and Practice	Insufficient data for extraction	10–19 y subgroup outcomes not reported
Farr (2021)	Feasibility of a randomized…	Disability and Rehabilitation	Insufficient data for extraction	10–19 y subgroup outcomes not reported
Fidan (2023)	Effect of virtual reality training…	Turkish Journal of Physiotherapy and Rehabilitation	Insufficient data for extraction	10–19 y subgroup outcomes not reported
Fluet (2010)	Interfacing a haptic robotic system…	Developmental Neurorehabilitation	Insufficient data for extraction	10–19 y subgroup outcomes not reported
Fu (2019)	Functional electrical stimulation…	Developmental Medicine and Child Neurology	Insufficient data for extraction	10–19 y subgroup outcomes not reported
Fu (2022)	Virtual reality combined with robot…	Technology and Health Care	Insufficient data for extraction	10–19 y subgroup outcomes not reported
Gagliardi (2018)	Immersive Virtual Reality to…	Annals of Biomedical Engineering	Insufficient data for extraction	10–19 y subgroup outcomes not reported
Gatica-Rojas (2017)	Does Nintendo Wii Balance Board…	European Journal of Physical and Rehabilitation Medicine	Insufficient data for extraction	10–19 y subgroup outcomes not reported
Gehringer (2023)	Hand-Arm Bimanual Intensive…	Pediatric Physical Therapy	Insufficient data for extraction	10–19 y subgroup outcomes not reported
Gercek (2022)	Alternative exercise methods for…	International Journal of Developmental Disabilities	Insufficient data for extraction	10–19 y subgroup outcomes not reported
Gordon (2012)	Potential of the Nintendo Wii TM…	Physiotherapy	Insufficient data for extraction	10–19 y subgroup outcomes not reported
Goyal (2022)	Non-Immersive Virtual Reality as an…	Cureus	Insufficient data for extraction	10–19 y subgroup outcomes not reported
Hamed (2022)	Effect of virtual reality games on…	Physiotherapy Quarterly	Insufficient data for extraction	10–19 y subgroup outcomes not reported
Harris (2005)	The influence of virtual reality play…	Revue Canadienne d Ergotherapie	Insufficient data for extraction	10–19 y subgroup outcomes not reported
Herzog (2017)	Advanced technologies…	Developmental Medicine and Child Neurology	Insufficient data for extraction	10–19 y subgroup outcomes not reported
Hobbs (2014)	The design of an accessible….	Developmental Medicine and Child Neurology	Insufficient data for extraction	10–19 y subgroup outcomes not reported
Holmström (2010)	Training of finger movement speed…	Developmental Medicine and Child Neurology	Insufficient data for extraction	10–19 y subgroup outcomes not reported
Hosseini (2025)	Combining nonlinear mechanical…	Biomedical Engineering Online	Insufficient data for extraction	10–19 y subgroup outcomes not reported

Hosseini (2023)	A New Training Protocol Based on…	Iranian Journal of Pediatrics	Insufficient data for extraction	10–19 y subgroup outcomes not reported
Hsieh (2020)	Preliminary Study of the Effect of…	American Journal of Physical Medicine and Rehabilitation	Insufficient data for extraction	10–19 y subgroup outcomes not reported
James (2015)	Randomized controlled trial of web…	Developmental Medicine and Child Neurology	Insufficient data for extraction	10–19 y subgroup outcomes not reported
Jaume-i-Capo (2014)	Interactive rehabilitation system for…	IEEE Transactions on Neural Systems and Rehabilitation Engineering	Insufficient data for extraction	10–19 y subgroup outcomes not reported
Jelsma (2011)	Nintendo WII-FIT; is it a useful…	Physiotherapy (United Kingdom)	Insufficient data for extraction	10–19 y subgroup outcomes not reported
Jelsma (2013)	The effect of the Nintendo Wii Fit…	Developmental Neurorehabilitation	Insufficient data for extraction	10–19 y subgroup outcomes not reported
Jha (2021)	Randomised trial of virtual reality…	Somatosensory and Motor Research	Insufficient data for extraction	10–19 y subgroup outcomes not reported
Jung (2022)	The Effect of a Horse-Riding…	Sensors	Insufficient data for extraction	10–19 y subgroup outcomes not reported
Kachmar (2017)	Stepping games for motor training…	Physiotherapy (United Kingdom)	Insufficient data for extraction	10–19 y subgroup outcomes not reported
Kachmar (2021)	Personalized balance games for…	Developmental Medicine and Child Neurology	Insufficient data for extraction	10–19 y subgroup outcomes not reported
Kachmar (2021)	Personalized balance games for…	Journal of Pediatric Rehabilitation Medicine	Insufficient data for extraction	10–19 y subgroup outcomes not reported
Kanitkar (2023)	Evaluation of a computer game…	PM & R	Insufficient data for extraction	10–19 y subgroup outcomes not reported
Keller (2017)	Weight-supported training of the…	Journal of Neuroengineering and Rehabilitation	Insufficient data for extraction	10–19 y subgroup outcomes not reported
Klobucka (2013)	Influence of virtual reality…	Journal of the Neurological Sciences	Insufficient data for extraction	10–19 y subgroup outcomes not reported
Knights (2013)	The impact of an interactive…	Developmental Medicine and Child Neurology	Insufficient data for extraction	10–19 y subgroup outcomes not reported
Knights (2014)	An innovative cycling exergame to…	Developmental Neurorehabilitation	Insufficient data for extraction	10–19 y subgroup outcomes not reported
Ko (2013)	Do virtual reality sports games…	Developmental Medicine and Child Neurology	Insufficient data for extraction	10–19 y subgroup outcomes not reported
Kozyavkin (2020)	Hand function in children with…	Developmental Medicine and Child Neurology	Insufficient data for extraction	10–19 y subgroup outcomes not reported
Lee (2012)	Improvement of throwing motion…	PM & R	Insufficient data for extraction	10–19 y subgroup outcomes not reported
Liechty (2025)	A Critically Appraised Paper on…	Journal of Hand Therapy	Insufficient data for extraction	10–19 y subgroup outcomes not reported
Lim (2025)	Preliminary Effects of Extended…	Bioengineering	Insufficient data for extraction	10–19 y subgroup outcomes not reported
Lopez-Ortiz (2012)	A pilot study using virtual reality…	Developmental Medicine and Child Neurology	Insufficient data for extraction	10–19 y subgroup outcomes not reported
Luna-Oliva (2013)	Kinect Xbox 360 as a therapeutic…	NeuroRehabilitation	Insufficient data for extraction	10–19 y subgroup outcomes not reported
Macintosh (2019)	MyoDashy: Building a game to…	Developmental Medicine and Child Neurology	Insufficient data for extraction	10–19 y subgroup outcomes not reported
MacIntosh (2021)	A Classification and Calibration…	IEEE Transactions on Neural Systems and Rehabilitation Engineering	Insufficient data for extraction	10–19 y subgroup outcomes not reported
Madboly (2024)	Virtual reality versus balance beam…	Fizjoterapia polska	Insufficient data for extraction	10–19 y subgroup outcomes not reported
Malick (2022)	Effects of Augmented Reality…	Frontiers in Neurology		

Menekseoglu (2023)	Effect of a Virtual Reality-Mediated…	American Journal of Physical Medicine and Rehabilitation	Insufficient data for extraction	10–19 y subgroup outcomes not reported
Metin Okmen (2019)	Effect of virtual reality therapy on…	Turkish Journal of Physical Medicine and Rehabilitation	Insufficient data for extraction	10–19 y subgroup outcomes not reported
Meyns (2019)	Exergaming does not improve…	Gait and Posture	Insufficient data for extraction	10–19 y subgroup outcomes not reported
Meyns (2022)	Exergaming improves balance in…	Disability and Rehabilitation	Insufficient data for extraction	10–19 y subgroup outcomes not reported
Meyns (2024)	The effectiveness of home-based…	Gait and Posture	Insufficient data for extraction	10–19 y subgroup outcomes not reported
Meyns (2017)	Can virtual reality games improve…	Gait and Posture	Insufficient data for extraction	10–19 y subgroup outcomes not reported
Mouhamed (2024)	Efficacy of virtual reality on balance…	European journal of physical & rehabilitation medicine	Insufficient data for extraction	10–19 y subgroup outcomes not reported
Ochandorena-Acha (2022)	Effectiveness of virtual reality on…	BMJ Open	Insufficient data for extraction	10–19 y subgroup outcomes not reported
Park (2021)	Effect of posture control training…	NeuroRehabilitation	Insufficient data for extraction	10–19 y subgroup outcomes not reported
Peramalaiah (2025)	Evaluation of a Game-Based…	JMIR Rehabilitation And Assistive Technologies	Insufficient data for extraction	10–19 y subgroup outcomes not reported
Pourazar (2021)	Virtual reality training improves…	International Journal of Developmental Disabilities	Insufficient data for extraction	10–19 y subgroup outcomes not reported
Pourazar (2018)	Use of virtual reality intervention to…	Developmental Neurorehabilitation	Insufficient data for extraction	10–19 y subgroup outcomes not reported
Preston (2016)	Feasibility of school-based…	Disability & Rehabilitation Assistive Technology	Insufficient data for extraction	10–19 y subgroup outcomes not reported
Ramstrand (2012)	Can balance in children with…	Technology and Health Care	Insufficient data for extraction	10–19 y subgroup outcomes not reported
Reid (2006)	The use of virtual reality with…	Therapeutic Recreation Journal	Insufficient data for extraction	10–19 y subgroup outcomes not reported
Rerkmoung (2024)	Comparative Study of Effects of…	Archives of Physical Medicine and Rehabilitation	Insufficient data for extraction	10–19 y subgroup outcomes not reported
Roberts (2025)	Constraint Therapy with and Without…	Children	Insufficient data for extraction	10–19 y subgroup outcomes not reported
Roostaei (2023)	Effects of a multi-component virtual…	Heliyon	Insufficient data for extraction	10–19 y subgroup outcomes not reported
Rostami (2012)	Effects of modified constraint…	NeuroRehabilitation	Insufficient data for extraction	10–19 y subgroup outcomes not reported
Rajesh S (2024)	Comparing the Effects of Virtual…	Cureus	Insufficient data for extraction	10–19 y subgroup outcomes not reported
Saad-Eldien (2025)	Efficacy of Plyometric Exercises…	NeuroRehabilitation	Insufficient data for extraction	10–19 y subgroup outcomes not reported
Sajan (2017)	Wii-based interactive video games…	Developmental Neurorehabilitation	Insufficient data for extraction	10–19 y subgroup outcomes not reported
Sandlund (2014)	Training of goal directed arm…	Developmental Neurorehabilitation	Insufficient data for extraction	10–19 y subgroup outcomes not reported
Sandlund (2011)	Using motion interactive games to…	Developmental Neurorehabilitation	Insufficient data for extraction	10–19 y subgroup outcomes not reported
Saussez (2023)	Efficacy of integrating a semi…	Journal of Neuroengineering and Rehabilitation	Insufficient data for extraction	10–19 y subgroup outcomes not reported
Selimoglu Ayneli (2025)	Comparison of the complementary…	Turkish Journal of Physical Medicine and Rehabilitation	Insufficient data for extraction	10–19 y subgroup outcomes not reported
Sharan (2012)	Virtual reality based therapy for post…	Sage Journals	Insufficient data for extraction	10–19 y subgroup outcomes not reported
Sharan (2018)	Two models of virtual reality devices…	Annals of Physical and Rehabilitation Medicine	Insufficient data for extraction	10–19 y subgroup outcomes not reported
Shih (2023)	Comparative effects of kinect-based…	Journal of Neuroengineering and Rehabilitation	Insufficient data for extraction	10–19 y subgroup outcomes not reported

Shum (2020)	Error Augmentation in Immersive…	IEEE Transactions on Neural Systems and Rehabilitation Engineering	Insufficient data for extraction	10–19 y subgroup outcomes not reported
Tarakci (2016)	Effects of Nintendo Wii-Fit…	Pediatrics International	Insufficient data for extraction	10–19 y subgroup outcomes not reported
Tarakci (2020)	Leap Motion Controller-based…	Journal of Hand Therapy	Insufficient data for extraction	10–19 y subgroup outcomes not reported
Tresser (2023)	Personalisation of a virtual gaming…	Disability & Rehabilitation Assistive Technology	Insufficient data for extraction	10–19 y subgroup outcomes not reported
Turconi (2016)	Can new technologies improve…	European journal of physical & rehabilitation medicine	Insufficient data for extraction	10–19 y subgroup outcomes not reported
Velasco (2017)	Evaluation of cervical posture…	Biomedical Engineering Online	Insufficient data for extraction	10–19 y subgroup outcomes not reported
Wang (2021)	Commercial Exergaming in Home…	OTJR: Occupation, Participation, & Health	Insufficient data for extraction	10–19 y subgroup outcomes not reported
Weightman (2011)	Home based computer-assisted…	Journal of Rehabilitation Medicine	Insufficient data for extraction	10–19 y subgroup outcomes not reported
Wu (2010)	Feedback-controlled passive…	Developmental Medicine and Child Neurology	Insufficient data for extraction	10–19 y subgroup outcomes not reported
Yoo (2017)	Augmented effects of EMG…	NeuroRehabilitation	Insufficient data for extraction	10–19 y subgroup outcomes not reported
Ziab (2024)	Effectiveness of virtual reality…	Journal of Pediatric Rehabilitation Medicine	Insufficient data for extraction	10–19 y subgroup outcomes not reported

Table S6. Full-text reports excluded, with primary reasons

Registry records were identified by registry platform and registration number; where no author was available, the registry identifier was used for unambiguous traceability.
