# Supplementary material for: Virtual Reality-Assisted Rehabilitation for Adolescents with Cerebral Palsy: A Systematic Review and Meta-Analysis
Source: J Clin Med. 2026 Aug 12;15(16):6248. doi: 10.3390/jcm15166248 (PMC13513917; doi:10.3390/jcm15166248)
Supplement: Supplementary file 1 [file jcm-15-06248-s001.zip › Supplementary_Table_S7_FullSearchStrategies.pdf]

Supplementary Table S7. Full electronic search strategies.

Note: Record counts reflect the number of records retrieved from each database before deduplication and screening.

Unless otherwise stated, searches were conducted from database inception to the search date, with no language, age, or publication-status limits applied. Age eligibility (10–19 years) and study design eligibility were applied during screening and data extraction.

Embase (Embase.com, Elsevier) — Date searched: 25 Feb 2026 — Records retrieved: 915

#	Search strategy (run as entered)
1	(((Cerebr* OR Brain* OR Centra*) NEAR/4 (Pals* OR Paralys* OR Pares*)) OR (encephalopath* NEAR/4 Infantal*) OR (Little* NEAR/2 disease*) OR ((Spasti* OR Dyskineti* OR Ataxi* OR Atoni* OR Hypotoni* OR Athetoi*) NEAR/4 (Monoplegi* OR Diplegi* OR Hemiplegi* OR Quadriplegi*))):ti,ab,kw
2	"cerebral palsy"/exp
3	((Virtual* NEAR/4 (Realit* OR System* OR Environment*)) OR ((Computer* OR Video* OR Television* OR TV* OR Motion-sens* OR Seriou*) NEAR/4 game*) OR Videogame* OR VR* OR Hardware* OR Exergame* OR Gamifi* OR ((Character* OR Command-line OR Graphic* OR Text*) NEAR/4 Interfac*) OR (Game* NEAR/4 (Rehabilitation* OR Therap*))):ti,ab,kw
4	"virtual reality"/exp OR "exergaming"/exp OR "computer interface"/exp OR "computer simulation"/exp OR "virtual reality exposure therapy"/exp
5	(#1 OR #2) AND (#3 OR #4) AND [embase]/lim

Fields: title/abstract/keyword (ti,ab,kw). Proximity operator: NEAR/n. Controlled vocabulary: Emtree (exp = explode).

MEDLINE (Ovid) — Date searched: 25 Feb 2026 — Records retrieved: 813

#	Search strategy (run as entered)
1	(((Cerebr* OR Brain* OR Centra*) ADJ4 (Pals* OR Paralys* OR Pares*)) OR (encephalopath* ADJ4 Infantal*) OR (Little* ADJ2 disease*) OR ((Spasti* OR Dyskineti* OR Ataxi* OR Atoni* OR Hypotoni* OR Athetoi*) ADJ4 (Monoplegi* OR Diplegi* OR Hemiplegi* OR Quadriplegi*))).ti,ab,kf
2	exp Cerebral Palsy/
3	((Virtual* ADJ4 (Realit* OR System* OR Environment*)) OR ((Computer* OR Video* OR Television* OR TV* OR Motion-sens* OR Seriou*) ADJ4 game*) OR Videogame* OR VR* OR Hardware* OR Exergame* OR Gamifi* OR ((Character* OR Command-line OR Graphic* OR Text*) ADJ4 Interfac*) OR (Game* ADJ4 (Rehabilitation* OR Therap*))).ti,ab,kf
4	exp Virtual Reality/ OR exp Exergaming/ OR exp User-Computer Interface/ OR exp Computer Simulation/ OR exp Virtual Reality Exposure Therapy/
5	(1 OR 2) AND (3 OR 4)

Fields: title/abstract/keyword heading word (ti,ab,kf). Proximity operator: ADJ/n. Controlled vocabulary: MeSH (exp = explode).

Cochrane CENTRAL (Cochrane Library) — Date searched: 25 Feb 2026 — Records retrieved: 376

#	Search strategy (run as entered)
1	(((Cerebr* OR Brain* OR Centra*) NEAR/4 (Pals* OR Paralys* OR Pares*)) OR (encephalopath* NEAR/4 Infantal*) OR (Little* NEAR/2 disease*) OR ((Spasti* OR Dyskineti* OR Ataxi* OR Atoni* OR Hypotoni* OR Athetoi*) NEAR/4 (Monoplegi* OR Diplegi* OR Hemiplegi* OR Quadriplegi*))):ti,ab,kw
2	[mh "Cerebral palsy"]
3	((Virtual* NEAR/4 (Realit* OR System* OR Environment*)) OR ((Computer* OR Video* OR Television* OR TV* OR Motion-sens* OR Seriou*) NEAR/4 game*) OR Videogame* OR VR* OR Hardware* OR Exergame* OR Gamifi* OR ((Character* OR Command-line OR Graphic* OR Text*) NEAR/4 Interfac*) OR (Game* NEAR/4 (Rehabilitation* OR Therap*))):ti,ab,kw
4	[mh "Virtual reality"] OR [mh "Exergaming"] OR [mh "User-computer interface"] OR [mh "Computer simulation"] OR [mh "Virtual reality exposure therapy"]
5	(#1 OR #2) AND (#3 OR #4)

CENTRAL is a trials register; no additional study-design filter was applied beyond database scope. Fields: title/abstract/keyword (ti,ab,kw).
