# Supplementary material for: Virtual Reality-Assisted Rehabilitation for Adolescents with Cerebral Palsy: A Systematic Review and Meta-Analysis
Source: J Clin Med. 2026 Aug 12;15(16):6248. doi: 10.3390/jcm15166248 (PMC13513917; doi:10.3390/jcm15166248)
Supplement: Supplementary file 1 [file jcm-15-06248-s001.zip › Supplementary_Table_S8_PRISMA_2020_Checklist.pdf]

PRISMA 2020 Checklist

Section and Topic	Item #	Checklist item	Location where item is reported
**TITLE**			
Title	1	Identify the report as a systematic review.	Title
**ABSTRACT**			
Abstract	2	See the PRISMA 2020 for Abstracts checklist.	Abstract
**INTRODUCTION**			
Rationale	3	Describe the rationale for the review in the context of existing knowledge.	Introduction
Objectives	4	Provide an explicit statement of the objective(s) or question(s) the review addresses.	Introduction
**METHODS**			
Eligibility criteria	5	Specify the inclusion and exclusion criteria for the review and how studies were grouped for the syntheses.	Methodology—Eligibility criteria
Information sources	6	Specify all databases, registers, websites, organisations, reference lists and other sources searched or consulted to identify studies. Specify the date when each source was last searched or consulted.	Methodology—Information sources and search strategy; date last searched: 25 Feb 2026
Search strategy	7	Present the full search strategies for all databases, registers and websites, including any filters and limits used.	Methodology—Information sources and search strategy; Supplementary Table S7
Selection process	8	Specify the methods used to decide whether a study met the inclusion criteria of the review, including how many reviewers screened each record and each report retrieved, whether they worked independently, and if applicable, details of automation tools used in the process.	Methodology—Study selection
Data collection process	9	Specify the methods used to collect data from reports, including how many reviewers collected data from each report, whether they worked independently, any processes for obtaining or confirming data from study investigators, and if applicable, details of automation tools used in the process.	Methodology—Data extraction
Data items	10a	List and define all outcomes for which data were sought. Specify whether all results that were compatible with each outcome domain in each study were sought (e.g. for all measures, time points, analyses), and if not, the methods used to decide which results to collect.	Methodology—Outcomes; Supplementary Table S5
	10b	List and define all other variables for which data were sought (e.g. participant and intervention characteristics, funding sources). Describe any assumptions made about any missing or unclear information.	Methodology—Data extraction; Table 1
Study risk of bias assessment	11	Specify the methods used to assess risk of bias in the included studies, including details of the tool(s) used, how many reviewers assessed each study and whether they worked independently, and if applicable, details of automation tools used in the process.	Methodology—Risk of bias assessment; Supplementary Table S1
Effect measures	12	Specify for each outcome the effect measure(s) (e.g. risk ratio, mean difference) used in the synthesis or presentation of results.	Methodology—Data synthesis and statistical analysis
Synthesis methods	13a	Describe the processes used to decide which studies were eligible for each synthesis (e.g. tabulating the study intervention characteristics and comparing against the planned groups for each synthesis (item #5)).	Methodology—Study selection
	13b	Describe any methods required to prepare the data for presentation or synthesis, such as handling of missing summary statistics, or data conversions.	Methodology—Data extraction; Supplementary Data 2; Supplementary Table S3
	13c	Describe any methods used to tabulate or visually display results of individual studies and syntheses.	Methodology—Data synthesis and statistical analysis; Figure 2; Figure 3; Supplementary Figures S1–S4

PRISMA 2020 Checklist

Section and Topic	Item #	Checklist item	Location where item is reported
	13d	Describe any methods used to synthesize results and provide a rationale for the choice(s). If meta-analysis was performed, describe the model(s), method(s) to identify the presence and extent of statistical heterogeneity, and software package(s) used.	Methodology—Data synthesis and statistical analysis
	13e	Describe any methods used to explore possible causes of heterogeneity among study results (e.g. subgroup analysis, meta-regression).	Methodology—Data synthesis and statistical analysis; Supplementary Figures S1–S2
	13f	Describe any sensitivity analyses conducted to assess robustness of the synthesized results.	Methodology—Data synthesis and statistical analysis; Supplementary Figure S3
Reporting bias assessment	14	Describe any methods used to assess risk of bias due to missing results in a synthesis (arising from reporting biases).	Methodology—Data synthesis and statistical analysis
Certainty assessment	15	Describe any methods used to assess certainty (or confidence) in the body of evidence for an outcome.	Methodology—Certainty of evidence (GRADE)
**RESULTS**			
Study selection	16a	Describe the results of the search and selection process, from the number of records identified in the search to the number of studies included in the review, ideally using a flow diagram.	Results—Study selection; Supplementary Figure 1
	16b	Cite studies that might appear to meet the inclusion criteria, but which were excluded, and explain why they were excluded.	Results—Study selection; Supplementary Table S6
Study characteristics	17	Cite each included study and present its characteristics.	Results—Study characteristics; Table 1
Risk of bias in studies	18	Present assessments of risk of bias for each included study.	Results—Risk of bias within studies; Figure 3; Supplementary Table S1
Results of individual studies	19	For all outcomes, present, for each study: (a) summary statistics for each group (where appropriate) and (b) an effect estimate and its precision (e.g. confidence/credible interval), ideally using structured tables or plots.	Table 1; Supplementary Table S2; Figure 2
Results of syntheses	20a	For each synthesis, briefly summarise the characteristics and risk of bias among contributing studies.	Results—Study characteristics; Results—Effects of interventions
	20b	Present results of all statistical syntheses conducted. If meta-analysis was done, present for each the summary estimate and its precision (e.g. confidence/credible interval) and measures of statistical heterogeneity. If comparing groups, describe the direction of the effect.	Results—Effects of interventions; Figure 2
	20c	Present results of all investigations of possible causes of heterogeneity among study results.	Results—Additional analyses; Supplementary Figures S1–S2
	20d	Present results of all sensitivity analyses conducted to assess the robustness of the synthesized results.	Results—Additional analyses; Supplementary Figure S3
Reporting biases	21	Present assessments of risk of bias due to missing results (arising from reporting biases) for each synthesis assessed.	Results—Additional analyses; Supplementary Figure S4
Certainty of evidence	22	Present assessments of certainty (or confidence) in the body of evidence for each outcome assessed.	Results—Certainty of evidence; Table 2
**DISCUSSION**			
Discussion	23a	Provide a general interpretation of the results in the context of other evidence.	Discussion—Principal findings; Discussion—Interpretation and

PRISMA

PRISMA 2020 Checklist

Section and Topic	Item #	Checklist item	Location where item is reported
			comparison with prior literature
	23b	Discuss any limitations of the evidence included in the review.	Discussion—Strengths and limitations
	23c	Discuss any limitations of the review processes used.	Discussion—Strengths and limitations
	23d	Discuss implications of the results for practice, policy, and future research.	Discussion—Clinical implications; Discussion—Future research
**OTHER INFORMATION**			
Registration and protocol	24a	Provide registration information for the review, including register name and registration number, or state that the review was not registered.	Methodology—Protocol and reporting guideline
	24b	Indicate where the review protocol can be accessed, or state that a protocol was not prepared.	Methodology—Protocol and reporting guideline
	24c	Describe and explain any amendments to information provided at registration or in the protocol.	Methodology—Information sources and search strategy; Discussion—Strengths and limitations
Support	25	Describe sources of financial or non-financial support for the review, and the role of the funders or sponsors in the review.	Title page—Funding
Competing interests	26	Declare any competing interests of review authors.	Title page—Conflicts of interest
Availability of data, code and other materials	27	Report which of the following are publicly available and where they can be found: template data collection forms; data extracted from included studies; data used for all analyses; analytic code; any other materials used in the review.	Title page—Data availability; Supplementary Data 1–2

PRISMA 2020 for Abstracts Checklist

Section and Topic	Item #	Checklist item	Reported (Yes/No)
**TITLE**			
Title	1	Identify the report as a systematic review.	Yes
**BACKGROUND**			
Objectives	2	Provide an explicit statement of the main objective(s) or question(s) the review addresses.	Yes
**METHODS**			
Eligibility criteria	3	Specify the inclusion and exclusion criteria for the review.	Yes
Information sources	4	Specify the information sources (e.g. databases, registers) used to identify studies and the date when each was last searched.	Yes
Risk of bias	5	Specify the methods used to assess risk of bias in the included studies.	Yes
Synthesis of results	6	Specify the methods used to present and synthesise results.	Yes
**RESULTS**			
Included studies	7	Give the total number of included studies and participants and summarise relevant characteristics of studies.	Yes
Synthesis of results	8	Present results for main outcomes, preferably indicating the number of included studies and participants for each. If meta-analysis was done, report the summary estimate and confidence/credible interval. If comparing groups, indicate the direction of the effect (i.e. which group is favoured).	Yes
**DISCUSSION**			
Limitations of evidence	9	Provide a brief summary of the limitations of the evidence included in the review (e.g. study risk of bias, inconsistency and imprecision).	Yes
Interpretation	10	Provide a general interpretation of the results and important implications.	Yes
**OTHER**			
Funding	11	Specify the primary source of funding for the review.	No
Registration	12	Provide the register name and registration number.	Yes

PRISMA-P (Preferred Reporting Items for Systematic review and Meta-Analysis Protocols) 2015 checklist: recommended items to address in a systematic review protocol*

Section and topic	Item No	Checklist item	Location / notes
**ADMINISTRATIVE INFORMATION**			
**Title:**			
Identification	1a	Identify the report as a protocol of a systematic review	Yes. Full protocol p.1 (title identifies protocol of a systematic review/meta-analysis).
Update	1b	If the protocol is for an update of a previous systematic review, identify as such	Not applicable. This is not an update of a previous systematic review protocol.
Registration	2	If registered, provide the name of the registry (such as PROSPERO) and registration number	Yes. Full protocol p.1; PROSPERO record: CRD420261326441.
**Authors:**			
Contact	3a	Provide name, institutional affiliation, e-mail address of all protocol authors; provide physical mailing address of corresponding author	Partly addressed via PROSPERO record (authors/contact details). Journal corresponding author may differ from PROSPERO contact author.
Contributions	3b	Describe contributions of protocol authors and identify the guarantor of the review	Partly addressed. Objectives and review roles are implicit in protocol; guarantor not explicitly stated.
Amendments	4	If the protocol represents an amendment of a previously completed or published protocol, identify as such and list changes; otherwise, state plan for documenting important protocol amendments	Yes. Full protocol p.5 (Section 11, Amendments).
**Support:**			
Sources	5a	Indicate sources of financial or other support for the review	No external financial support stated in protocol or registry.
Sponsor	5b	Provide name for the review funder and/or sponsor	Not applicable / no named sponsor stated.
Role of sponsor or funder	5c	Describe roles of funder(s), sponsor(s), and/or institution(s), if any, in developing the protocol	Not applicable / no sponsor or funder role stated.
**INTRODUCTION**			
Rationale	6	Describe the rationale for the review in the context of what is already known	Yes. Full protocol p.1 (Section 1, Background and rationale).
Objectives	7	Provide an explicit statement of the question(s) the review will address with reference to participants, interventions, comparators, and outcomes (PICO)	Yes. Full protocol pp.1–2 (Section 2, Objectives).
**METHODS**			

Eligibility criteria	8	Specify the study characteristics (such as PICO, study design, setting, time frame) and report characteristics (such as years considered, language, publication status) to be used as criteria for eligibility for the review	Yes. Full protocol pp.2–3 (Section 3, Eligibility criteria).
Information sources	9	Describe all intended information sources (such as electronic databases, contact with study authors, trial registers or other grey literature sources) with planned dates of coverage	Partly addressed. Full protocol p.3 lists Embase, MEDLINE, and CENTRAL; PROSPERO record additionally lists citation searching and other sources.
Search strategy	10	Present draft of search strategy to be used for at least one electronic database, including planned limits, such that it could be repeated	Partly addressed. Protocol p.3 refers to an accompanying document containing full database search strategies; see Supplementary Table S7.
**Study records:**			
Data management	11a	Describe the mechanism(s) that will be used to manage records and data throughout the review	Yes. Full protocol pp.3–4 (record export/deduplication; extraction and management).
Selection process	11b	State the process that will be used for selecting studies (such as two independent reviewers) through each phase of the review (that is, screening, eligibility and inclusion in meta-analysis)	Yes. Full protocol p.3 (two independent reviewers; disagreement resolution).
Data collection process	11c	Describe planned method of extracting data from reports (such as piloting forms, done independently, in duplicate), any processes for obtaining and confirming data from investigators	Yes. Full protocol pp.3–4 (piloted extraction form; duplicate extraction planned).
Data items	12	List and define all variables for which data will be sought (such as PICO items, funding sources), any pre-planned data assumptions and simplifications	Yes. Full protocol pp.3–4 (study, intervention, comparator, and outcome data items).
Outcomes and prioritization	13	List and define all outcomes for which data will be sought, including prioritization of main and additional outcomes, with rationale	Yes. Full protocol pp.2–3 (primary and secondary outcomes prespecified and prioritised).
Risk of bias in individual studies	14	Describe anticipated methods for assessing risk of bias of individual studies, including whether this will be done at the outcome or study level, or both; state how this information will be used in data synthesis	Yes. Full protocol p.4 (Section 7, design-appropriate risk-of-bias tools).
Data synthesis	15a	Describe criteria under which study data will be quantitatively synthesised	Yes. Full protocol p.4 (criteria for quantitative synthesis: comparable outcomes and ≥2 studies).
	15b	If data are appropriate for quantitative synthesis, describe planned summary measures, methods of handling data and methods of combining data from studies, including any planned exploration of consistency (such as I^2, Kendall's τ)	Yes. Full protocol p.4 (random-effects meta-analysis, Hedges' g, 95% CI, handling of intervention-group pre–post data, heterogeneity by I^2).
	15c	Describe any proposed additional analyses (such as sensitivity or subgroup analyses, meta-regression)	Yes. Full protocol pp.4–5 (subgroup analyses, leave-one-out sensitivity analyses, additional sensitivity analyses).

	15d	If quantitative synthesis is not appropriate, describe the type of summary planned	Partly addressed. If meta-analysis is not appropriate, narrative synthesis is implied but not described in detail.
Meta-bias(es)	16	Specify any planned assessment of meta-bias(es) (such as publication bias across studies, selective reporting within studies)	Yes. Full protocol p.4–5 (Section 8.3, reporting bias assessment).
Confidence in cumulative evidence	17	Describe how the strength of the body of evidence will be assessed (such as GRADE)	Yes. Full protocol p.5 (Section 9, GRADE).

*** It is strongly recommended that this checklist be read in conjunction with the PRISMA-P Explanation and Elaboration (cite when available) for important clarification on the items. Amendments to a review protocol should be tracked and dated. The copyright for PRISMA-P (including checklist) is held by the PRISMA-P Group and is distributed under a Creative Commons Attribution Licence 4.0.**

PRISMA-S Checklist

Section/topic	#	Checklist item	Location(s) Reported
**INFORMATION SOURCES AND METHODS**			
Database name	1	Name each individual database searched, stating the platform for each.	Methodology—Information sources and search strategy; Supplementary Table S7.
Multi-database searching	2	If databases were searched simultaneously on a single platform, state the name of the platform, listing all of the databases searched.	N/A (Searched separately).
Study registries	3	List any study registries searched.	Not searched.
Online resources and browsing	4	Describe any online or print source purposefully searched or browsed (e.g., tables of contents, print conference proceedings, web sites), and how this was done.	N/A (no additional online or print sources were purposefully browsed beyond citation searching).
Citation searching	5	Indicate whether cited references or citing references were examined, and describe any methods used for locating cited/citing references (e.g., browsing reference lists, using a citation index, setting up email alerts for references citing included studies).	Methodology—Information sources and search strategy (backward/forward reference checking undertaken; no additional eligible studies identified).
Contacts	6	Indicate whether additional studies or data were sought by contacting authors, experts, manufacturers, or others.	N/A (no author, expert, manufacturer, or other contact).
Other methods	7	Describe any additional information sources or search methods used.	N/A (no additional search methods).
**SEARCH STRATEGIES**			
Full search strategies	8	Include the search strategies for each database and information source, copied and pasted exactly as run.	Supplementary Table S7.
Limits and restrictions	9	Specify that no limits were used, or describe any limits or restrictions applied to a search (e.g., date or time period, language, study design) and provide justification for their use.	Methodology—Information sources and search strategy; Supplementary Table S7 (no language or publication-status limits were applied in the electronic searches; age and study-design eligibility criteria were

			applied during screening and data extraction).
Search filters	10	Indicate whether published search filters were used (as originally designed or modified), and if so, cite the filter(s) used.	N/A (no search filters were used).
Prior work	11	Indicate when search strategies from other literature reviews were adapted or reused for a substantive part or all of the search, citing the previous review(s).	N/A.
Updates	12	Report the methods used to update the search(es) (e.g., rerunning searches, email alerts).	N/A (no search updates after 25 February 2026).
Dates of searches	13	For each search strategy, provide the date when the last search occurred.	Methodology—Information sources and search strategy (last searched 25 February 2026); Supplementary Table S7.
**PEER REVIEW**			
Peer review	14	Describe any search peer review process.	N/A (no PRESS/peer review of the electronic search strategy).
**MANAGING RECORDS**			
Total Records	15	Document the total number of records identified from each database and other information sources.	Results—Study selection; Figure 1 and Supplementary Table S7 (records retrieved per database: Embase 915; MEDLINE 813; CENTRAL 376).
Deduplication	16	Describe the processes and any software used to deduplicate records from multiple database searches and other information sources.	Methodology—Study selection: records were imported into EndNote for deduplication using title/author/year/DOI field matching, followed by manual review.

PRISMA-S: An Extension to the PRISMA Statement for Reporting Literature Searches in Systematic Reviews
