# Supplementary material for: Virtual Reality-Assisted Rehabilitation for Adolescents with Cerebral Palsy: A Systematic Review and Meta-Analysis
Source: J Clin Med. 2026 Aug 12;15(16):6248. doi: 10.3390/jcm15166248 (PMC13513917; doi:10.3390/jcm15166248)
Supplement: Supplementary file 1 [file jcm-15-06248-s001.zip › Supplementary_Table_S9_r_sensitivity_analysis.pdf]

Supplementary Table S9. Sensitivity analysis using alternative imputed within-participant pre–post correlation coefficients.

Scenario	Imputed r	Lower-limb/balance Hedges' g	Lower-limb/balance 95% CI	Upper-limb Hedges' g	Upper-limb 95% CI	Overall Hedges' g	Overall 95% CI	Fixed-effect Q_between (df = 1)	Interpretation
Sensitivity A	0.25	0.171	-0.194 to 0.537	0.458	0.105 to 0.812	0.319	0.037 to 0.600	Q = 5.393; p = 0.020	Interpretation unchanged
Primary analysis	0.50	0.209	-0.167 to 0.584	0.468	0.091 to 0.844	0.338	0.072 to 0.603	Q = 5.375; p = 0.020	Primary analysis
Sensitivity B	0.75	0.318	-0.086 to 0.722	0.486	0.057 to 0.914	0.397	0.103 to 0.691	Q = 5.335; p = 0.021	Interpretation unchanged

Values are based on CMA subgroup analyses using the functional-domain subgroup structure applied in the primary forest plot. Random-effects estimates are reported for pooled effects. The fixed-effect Q_between test was used for the subgroup difference test.

Only Jung et al. (2021) and Manikowska et al. (2021) required imputed pre-post correlations; empirically derived correlations for the remaining studies were retained across scenarios.

CI, confidence interval; CMA, Comprehensive Meta-Analysis; r, within-participant pre-post correlation coefficient.
